# Impact of METTL3-mediated m6A regulation on early human beige adipogenesis from hiPSCs

**DOI:** 10.3389/fcell.2026.1918478

**Published:** 2026-09-11

**Authors:** Sanjana Chandran, Moni Nader, Abdulrahim Sajini

**Affiliations:** 1 Department of Biomedical Engineering and Biotechnology, Khalifa University of Science and Technology, Abu Dhabi, United Arab Emirates; 2 Department of Medical Sciences, Khalifa University of Science and Technology, Abu Dhabi, United Arab Emirates; 3 Department of Physiological Sciences, College of Medicine, Alfaisal University, Riyadh, Saudi Arabia; 4 Department of Biology, Chemistry and Environmental Sciences, American University of Sharjah, Sharjah, United Arab Emirates

**Keywords:** beige adipogenesis, epitranscriptome, human induced pluripotent stem cells, N6-methyladenosine, METTL3, STM2457, UZH2

## Abstract

N6-methyladenosine (m6A) is a key post-transcriptional regulator of mammalian mRNA metabolism, yet its molecular role in human adipogenesis remains poorly understood. Using transgene-free human iPSC derived from adult (HDFa) and neonatal (HDFn) fibroblasts, we investigated the role of m6A during early adipocyte specification. We found that transient inhibition of METTL3 during the commitment phase (days 0–4) with STM2457 or UZH2 elicited distinct transcriptional and epitranscriptomic responses between the two human iPSC lines. Specifically, STM2457 reduced global m6A levels and impaired both adipogenic and thermogenic maturation in HDFa-derived iPSCs. Conversely, HDFn-derived iPSCs demonstrated better transcriptional plasticity, partially maintaining beige programs despite sustained m6A depletion. UZH2 yielded more variable outcomes, with more pronounced differentiation-associated alterations in the HDFa-derived line. These findings highlight a strong context-dependency in m6A regulation during adipogenesis. Collectively, our data demonstrate that METTL3-mediated m6A regulation acts as a flexible, time-sensitive coordinator of beige fat development rather than a uniform determinant of cell fate. Furthermore, the divergent responses observed between the two iPSC lines suggest that cellular context influences epitranscriptomic regulation during adipogenesis, reflecting contributions from donor origin, epigenetic state, and clonal variability, and warranting validation across additional independent iPSC lines. This study establishes a framework for investigating m6A biology in human adipogenesis and provides critical insights for modeling metabolic diseases.

## 1 Introduction

Obesity, a chronic disease caused by excessive fat accumulation, is a global health concern due to its rising prevalence and its role in conditions like type 2 diabetes, cardiovascular diseases, and several types of cancer (Frayling et al., 2007; Lan et al., 2020). Adipose tissue, made of adipocytes, plays a central role in energy homeostasis. There are three main adipocyte types: white (WA), brown (BA), and beige. WA stores energy as triglycerides, while BA and beige adipocytes are thermogenic, dissipating energy as heat. Beige adipocytes originate from WA through environmental stimuli and exhibit reversible thermogenic properties (Van Nguyen et al., 2020; Wu et al., 2012). The process of browning permits the conversion of white adipocytes into beige adipocytes, which are relatively more metabolically active. The unique plasticity and inducibility of beige adipocytes make them a targetable cell type to comprehend various factors involved in adipogenesis (Kononova et al., 2024; Peng et al., 2024). Adipogenesis, the process of fat cell formation, involves determination and terminal differentiation. This is not exclusive to obesity but is also relevant for broader disease models (Bahmad et al., 2020). The white-brown hybrid gene expression profile of beige adipocytes renders them a unique model to study cell fate decisions, epigenetics, and transcriptional regulation during adipogenesis (Boychenko et al., 2024; Shapira and Seale, 2019).

One promising approach to combating obesity is promoting the browning of WA. The RNA modification N6-methyladenosine (m6A), the most abundant mRNA modification in eukaryotes, has emerged as a critical regulator of adipogenesis. It affects mRNA splicing, stability, and translation through methyltransferases (“writers” such as METTL3, METTL14, and METTL16), demethylases (“erasers” such as FTO and ALKBH5), and binding proteins (“readers” such as YTHDFs and IGF2BPs) (Chen et al., 2019; Jiang et al., 2021). METTL3/METTL14 heterodimers catalyze m6A with assistance from cofactors like WTAP, ZC3H13, RBM15 and VIRMA (Shen et al., 2025). m6A is essential for stem cell differentiation, though its role in maintaining or disrupting pluripotency remains debated due to differences in stem cell types used (naive or primed). It was reported that in adipogenesis, m6A modulates key regulators such as JAK1 and C/EBPβ, influencing fat cell development (Wu and Wang, 2021).

Brown adipose tissue (BAT), prevalent in infants and declining with age, is a promising therapeutic target due to its thermogenic properties. Cell-based approaches using induced pluripotent stem cell (iPSC)-derived BA may supplement BAT in obese individuals. However, efficient, non-invasive, and scalable differentiation protocols are needed. iPSCs serve as a valuable model for studying adipogenesis and developing cell-based therapies due to their self-renewing and pluripotent nature (Takahashi and Yamanaka, 2006). Despite progress, protocols for differentiating iPSCs into functional adipocytes, especially beige types, are limited and often involve genetic modification which hinders clinical translation. Recent studies have achieved beige adipocyte differentiation from human induced pluripotent stem cells (hiPSCs) without transgene use, using mesodermal and adipogenic induction stages (Butts et al., 2021; Guénantin et al., 2017). However, challenges like epigenetic memory from source tissues and low adipogenic potential remain (Hafner et al., 2016; Poetsch et al., 2022).

**FIGURE 1:**
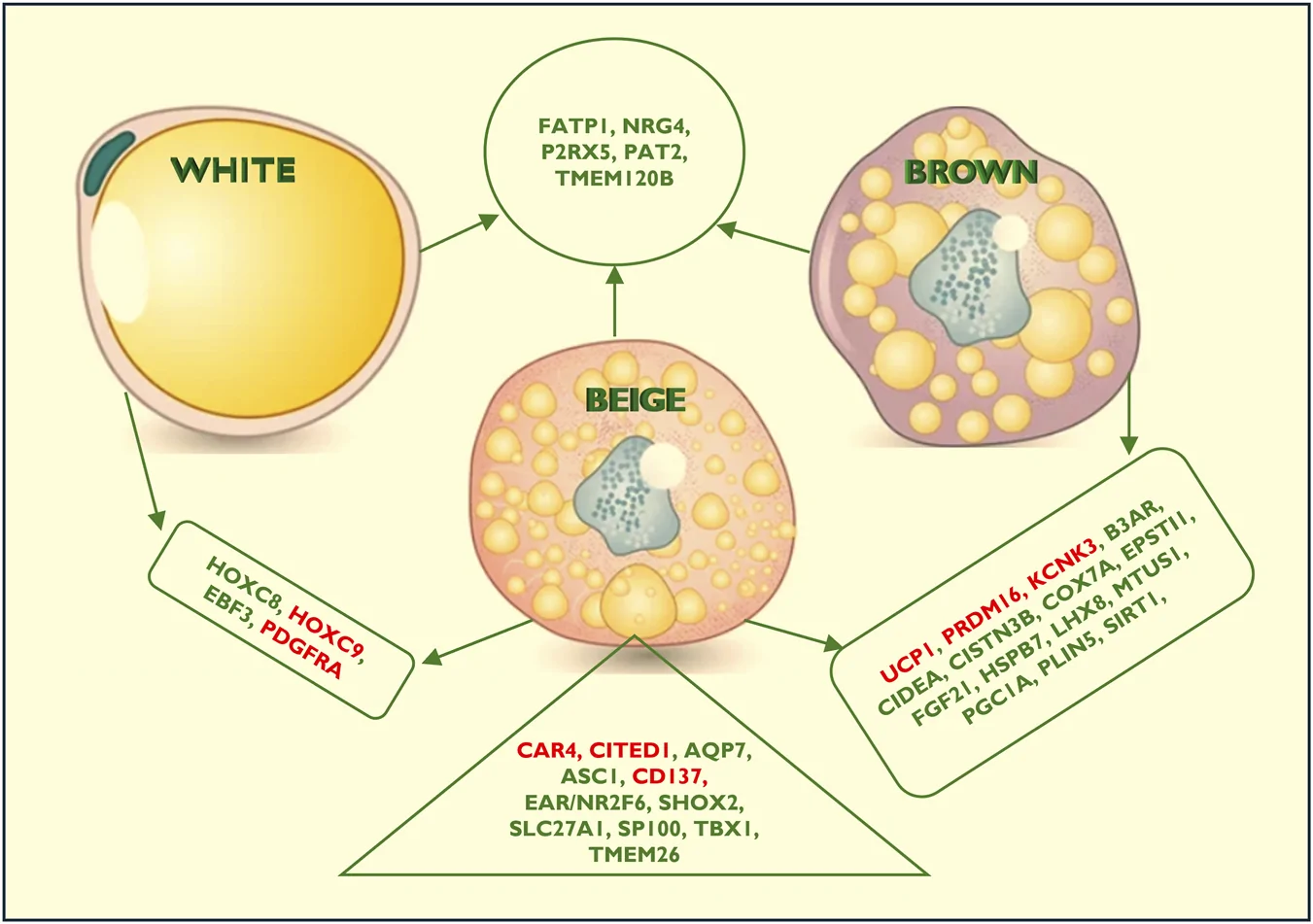
Beige adipocyte-associated genes that are unique or common with WA and BA.

Many factors appear to be highly determinative of BA phenotype and gene expression. PRDM16, PGC-1α, and PPARγ facilitate the acquisition, whereas transcription factors PRDM16, CEBPβ, EHMT1, ZFP516, and EWS regulate it, These mediate the differentiation of BA progenitors to mature adipocytes (Mohsen-Kanson et al., 2014). C/EBP family of proteins (C/EBPα, C/EBPβ, C/EBPδ), PRDM16 and PPARγ are claimed to play crucial roles in BA development, function, and maintenance (Ahfeldt et al., 2012; Van Nguyen et al., 2020). PR domain containing 16 (PRDM16) enacts a switch between skeletal muscle and BA. Notably, PRDM16 can stimulate brown-like characteristics before or during adipocyte differentiation. PRDM16 is present at very low levels in white adipocyte tissues (Van Nguyen et al., 2020). On the other hand, PPARγ, Hocx9, and Plin are markers found in WA. Whereas CD137, Tmem26, and CD40 are factors among beige adipocyte (Carobbio et al., 2019). CD29, PDGFRα, and CD44 are expressed in both white and beige adipocyte progenitors (Van Nguyen et al., 2020). The markers associated with beige adipocytes specifically and those that are reported to be found in common to beige-white or beige-brown types are given in Figure 1. Various research is underway to identify compounds and interventions that can stimulate the formation and activity of beige adipocytes, thereby harnessing their thermogenic and metabolic benefits for clinical applications. The most prominent genes associated with beige adipogenesis, whose roles are understood, are listed in Table 1.

**TABLE 1:** The markers associated with beige adipogenesis investigated in this study.

Gene	Role in beige adipogenesis	References
CEBPA	Master regulator of adipogenesis; influences and maintains adipocyte identity	(Rosen, 2005)
PPARG	Master regulator; promotes lipid accumulation and supports thermogenic programs in cooperation with beige/brown cofactors	(Rosen, 2005)
PRDM16	Drives beige/brown adipocyte fate; promotes UCP1 and mitochondrial biogenesis	(Jiang, N. et al., 2022)
CITED1	Beige-specific marker associated with thermogenic adipocyte identity	(Miller et al., 2015; Ziqubu et al., 2023)
HOXC9	Developmental transcription factor associated with beige adipocyte identity	(Miller et al., 2015; Ziqubu et al., 2023)
KCNK3	Ion channel enriched in beige adipocytes; regulates membrane potential	(Guénantin et al., 2017; Chen, Y. et al., 2017)
CAR4	Carbonic anhydrase; involved in pH regulation; marker of beige adipocytes	(Garcia et al., 2016)
UCP1	Mitochondrial uncoupling protein; hallmark of thermogenic adipocytes	(Ikeda and Yamada, 2020)
CD137	Surface marker enriched in beige adipocytes; recently been reported to suppress browning, requiring context-dependent interpretation	(Srivastava et al., 2020; Wu, J. et al., 2012)

Although information on the developmental origins of adipocytes in animal models is emerging, the lack of such information in humans calls for urgent investigation. Variations in m6A levels and the expression of their regulators have been observed at each stage of adipogenesis. We focused on the role of m6A modifications, specifically the writer METTL3, during mesodermal induction of beige adipogenesis from hiPSCs. The small molecules STM2457 and UZH2 are selective METTL3 inhibitors. STM2457 has been reported to block leukemic growth (Yankova et al., 2021). UZH2 demonstrated the ability to selectively target METTL3, thereby impairing proliferation in cancer cells (Dolbois et al., 2021). This study explores the impact of METTL3 inhibition employing STM2457 and UZH2 on m6A levels during mesodermal induction of beige adipogenesis from hiPSCs. A better understanding of m6A regulation in human adipogenesis could inform novel therapeutic strategies for obesity and metabolic diseases.

Adipogenesis is a tightly regulated, time-sensitive process in which the early commitment phase determines the final lineage trajectory. During the early stages of differentiation, cells undergo rapid transcriptional reprogramming and exit their progenitor state, activating core regulators that finalize their adipocyte identity. Hence, even short disruptions during this critical window can have profound, long-lasting effects on maturation (Kajimura et al., 2010; Rosen and Spiegelman, 2014). The m6A epitranscriptomic mark is vital during these early fate transitions, as it facilitates the timely degradation of pluripotency mRNAs and the coordinated expression of lineage-specific genes. Previous research suggests that METTL3-mediated m6A deposition is most critical during these early stages, with cells becoming less sensitive to m6A changes once lineage commitment is established (Geula et al., 2015; Wang et al., 2014). Consequently, this study restricted METTL3 inhibition to the commitment phase (days 0–4) to investigate its role in lineage decision-making (Yankova et al., 2021). This approach distinguishes the fundamental epitranscriptomic requirements of early differentiation from secondary effects or terminal maturation. By limiting inhibitor exposure, the study also minimized prolonged pharmacological stress, enabling a better assessment of how transient m6A disruption affects downstream adipogenic and thermogenic potential.

## 2 Materials and methods

### 2.1 Culturing human dermal fibroblasts

Adult and neonatal human dermal fibroblasts (HDFa and HDFn; Invitrogen) were maintained in Minimum Essential Medium supplemented with 20% Fetal bovine serum at 37 °C and 5% CO_2_. Cells were passaged at approximately 70%–80% confluence using 0.05% trypsin-EDTA and were routinely monitored for morphology and contamination.

### 2.2 Reprogramming fibroblasts to hiPSCs

Both types of fibroblasts were reprogrammed using the CytoTune 2.0 Sendai reprogramming vectors (Invitrogen) using feeder-free conditions. The kit protocol was followed by adding the reprogramming mixture to the cells and growing them for 7 days of transduction. Cells were seeded onto Matrigel-coated (Corning) 6-well plates post-transduction and grown in ReproTeSR (Stem Cell Technologies). Around the 21st day of growth, the colonies were manually picked and transferred to Matrigel-coated 6-well plates and grown in mTeSR™1 (Stem Cell Technologies). Furthermore, the colony passaging was acquired through ReLeSR (Stem Cell Technologies) (Orsi et al., 2024; Azzam et al., 2022).

### 2.3 Sendai transgenes clearance

The primer clearance checks for primers specific to Sendai transgenes, such as SeV, KOS, Klf, and c-Myc, were conducted by RT-PCR followed by electrophoresis. This was accomplished using GoTaq Green Master Mix (Promega). The transduced cells collected 1 week post-transduction were used as a positive control for vector detection (Matthes et al., 2025). The primer sequences chosen (Azzam et al., 2022) are given in Supplementary Table S1.

### 2.4 iPSC characterization

Total RNA was isolated using Trizol reagent (Invitrogen), and cDNA was synthesized using High- Capacity cDNA Reverse Transcription Kit (Applied Biosystems) according to the manufacturer’s instructions. qPCR was performed using SYBR green chemistry on a micPCR system (Bio Molecular Systems). Relative expression was calculated using the comparative Ct method with GAPDH as the reference gene (Azzam et al., 2022). Primer sequences are provided in Supplementary Table S1.

The iPSCs were differentiated into all three germ layers (ectoderm, endoderm, and Mesoderm) using the STEMdiff Trilineage Differentiation Kit (Stem Cell Technologies) according to the kit protocol (Rajendran et al., 2025). Briefly, hiPSCs were dissociated into single cells with Gentle Cell Dissociation Reagent (Stem Cell Technologies) and counted using Trypan Blue exclusion and a hemocytometer. Single cells were then seeded onto Matrigel-coated 24-well plates in mTeSR1 medium supplemented with 10 μM Y-27632, following the manufacturer’s recommended seeding densities: 400,000 cells for ectoderm, 100,000 cells for Mesoderm, and 400,000 cells for endoderm. Differentiation was carried out for 7 days in the ectoderm and 5 days in both the mesoderm and endoderm lineages (Veleva et al., 2024).

The iPSCs were fixed using 4% paraformaldehyde for 10 min at RT. 0.1% Triton X-100 in PBS was utilized to permeabilize the cells for 10 min. 1% BSA, 22.52 mg/mL glycine in PBS with 0.1% Tween-20 served as a blocking agent. Following incubation with primary/secondary antibodies, the cells were washed with PBS and counterstained with DAPI for nuclear staining. Pluripotency markers OCT4, TRA-1-60, and SSEA4 antibodies from Stem Cell Technologies were used to analyze (Azzam et al., 2022). The antibodies used for this are listed in the Supplementary Table S2. Lastly, immunocytochemistry was used to evaluate differentiation into the three germ layers as well. The table also lists the protein markers utilized for each germ layer. Alkaline phosphatase (ALP) is one of the key markers for identifying pluripotent embryonic stem cells and related cells (Romero-Vázquez et al., 2025). The iPSC colonies were fixed using 4% PFA and subjected to alkaline phosphatase staining using the kit from Merck. When hiPSCs reached approximately 70% confluence, ALP activity was evaluated using the detection kit following the manufacturer’s instructions. After a final wash with TBST, the cells were maintained in 1X PBS and imaged using an inverted microscope (ZEISS Axio Vert. A1, Germany).

Genomic DNA was isolated from the cultured hiPSCs using the Purelink genomic DNA Kit. Chromosomal stability was assessed with the hPSC Genetic Analysis Kit (Stemcell Technologies). Briefly, genomic DNA was combined with double-quenched primer-probe sets labeled with 5-FAM. These assays are designed to detect the eight most frequently reported karyotypic abnormalities in hiPSCs, including gains in chr 1q, 8q, 10p, 12p, 17q, 18q, 20q, and Xp. Quantitative PCR was performed using a QuantStudio 7 Flex system. Relative copy number variations were calculated using the ΔΔCt method and normalized to the chr 4p locus as an internal control, in accordance with the manufacturer’s instructions (Clayton et al., 2021; Chen et al., 2021). The genomic integrity of the PSC lines was assessed using the genetic analysis app offered by Stem cell technologies. Both HDFa and HDFn-derived iPSC lines showed recurrent copy number variations commonly reported in cultured human iPSCs, including low-level gains on chromosomes 8q and 20q. The report is given in Supplementary Figure S2. The MycoAlert Kit (Lonza) was used at regular intervals for quick and convenient detection of viable *mycoplasma* in cell cultures. Frequent testing, such as this, indicates when a cell line becomes infected, allowing prompt remedial action to be taken (Matthes et al., 2025).

### 2.5 Beige adipocyte differentiation and METTL3 inhibitor treatment

**FIGURE 2:**
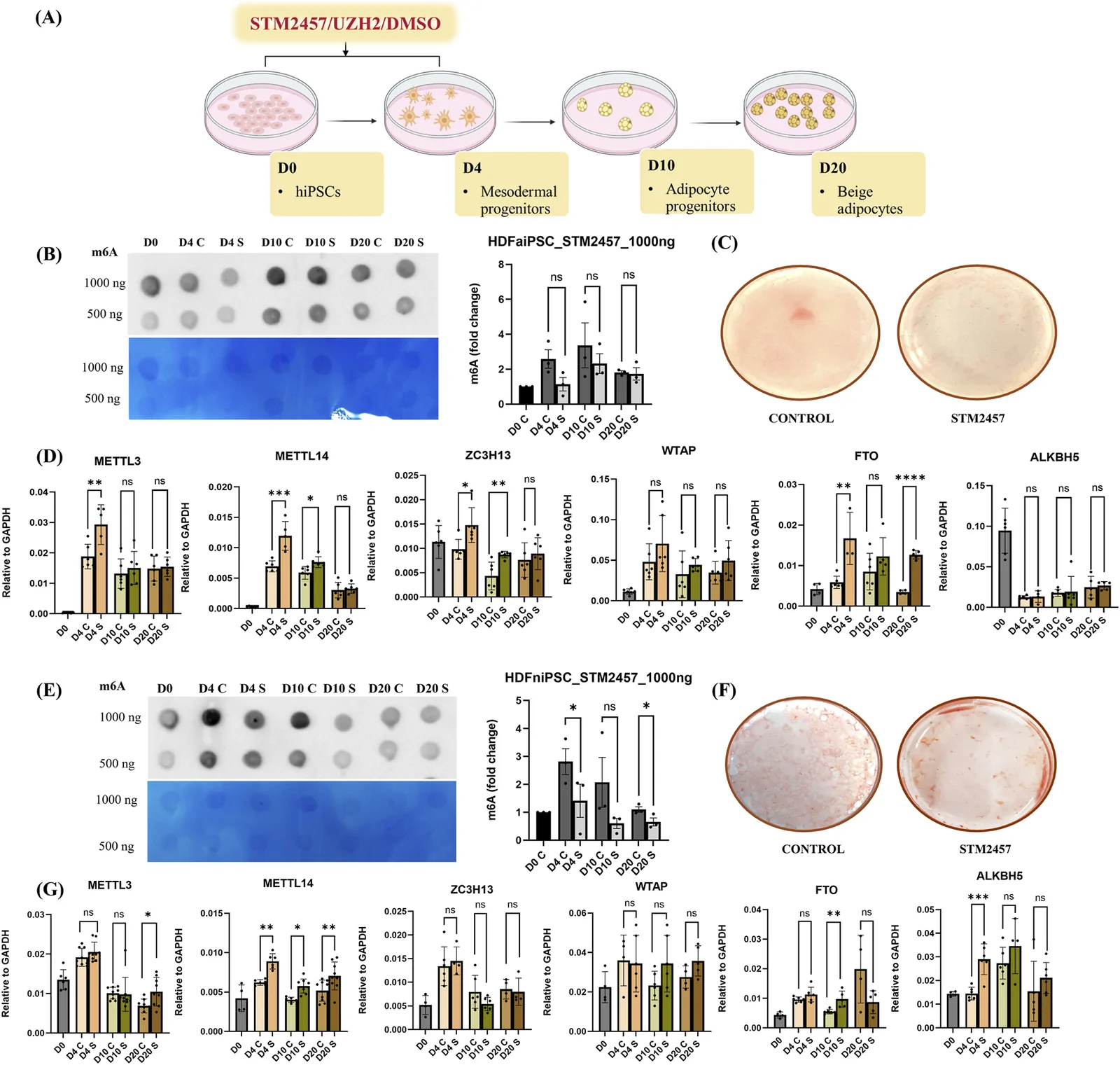
Effects of transient STM2457 treatment during beige adipogenesis. **(A)** Schematic of the 20-day beige adipocyte differentiation protocol and METTL3-inhibitor exposure from day 0 to day 4. **(B)** Representative m6A RNA dot blot and corresponding methylene-blue loading control for HDFa-iPSCs, with quantification of normalized m6A abundance. **(C)** Whole-well Oil Red O staining of control and STM2457-treated HDFa-derived cells at day 20. **(D)** Time-course expression of m6A regulatory genes in HDFa-iPSCs. **(E)** Representative m6A RNA dot blot and corresponding methylene-blue loading control for HDFn-iPSCs, with quantification of normalized m6A abundance. **(F)** Whole-well Oil Red O staining of control and STM2457-treated HDFn-derived cells at day 20. **(G)** Time-course expression of m6A regulatory genes in HDFn-iPSCs. C, control; S, STM2457. For m6A analysis, data represent mean ± SEM from three independent dot-blot experiments after normalization to the corresponding methylene-blue signal; quantification shown corresponds to the 1000-ng RNA spots. Matched control and STM2457-treated samples from the independent experiments were compared at each time point using a paired t-test. For qPCR **(D,G)**, individual points show technical duplicate measurements from three independent wells per condition. Control and STM2457-treated groups were compared at each time point using a two-tailed unpaired t-test. Data are presented as mean ± SD. Statistical significance is indicated as *p < 0.05, **p < 0.01, ***p < 0.001, ****p < 0.0001; ns, not significant.

This study investigated the role of m6A RNA methylation in beige adipogenesis by targeting the m6A methyltransferase METTL3 using small-molecule inhibitors. Two distinct inhibitors, STM2457 and UZH2, were introduced at early stages of differentiation to assess their impact on m6A dynamics and adipogenic commitment. Induced pluripotent stem cells (iPSCs) generated from adult (HDFa-iPSC) and neonatal fibroblasts (HDFn-iPSC) were utilized in separate experiments. Both lines were subjected to a 20-day beige adipocyte differentiation protocol (Guénantin et al., 2017), during which cells were sequentially transitioned through mesodermal progenitors, adipocyte progenitors, and ultimately mature beige adipocytes. The differentiation and inhibitor-treatment workflow is shown in Figure 2A.

To examine the effect of METTL3 inhibition, STM2457, a potent and selective small-molecule inhibitor of METTL3, was applied at the mesodermal induction stage (Day 0 to Day 4) at a concentration of 5 µM (Dong et al., 2023). Differentiation continued according to the protocol (Guénantin et al., 2017). Vehicle-treated controls using DMSO were processed in parallel under identical conditions. Mesoderm induction was performed using GlutaMAX, 50 μg/mL ascorbic acid, 10 ng/mL bone morphogenetic protein-4, and 25 ng/mL activin A. Adipogenic induction followed with DMEM/F12 supplemented with 10% FBS, 10 μg/mL insulin, 500 μmol/L isobutylmethylxanthine, 1 μmol/L dexamethasone, and 50 μmol/L indomethacin. Final adipocyte maturation occurred in DMEM/F12 with 10% FBS and 1 μg/mL insulin.

In a parallel set of experiments, UZH2, another METTL3 inhibitor, was used instead of STM2457 under otherwise identical conditions. To be precise, during mesodermal induction (Day 0-Day 4), the cells were subjected to the inhibitor at a concentration of 10 µM (Dolbois et al., 2021), and the differentiation progressed as per the protocol until day 20. In both cases, differentiation was monitored morphologically and molecularly. Differentiation was characterized at multiple stages (Days 0, 4, 10, and 20) through gene expression and immunofluorescence analyses. Cells were harvested in triplicate from three independent wells of a 6-well plate per condition for gene expression profiling. Additional wells were fixed using paraformaldehyde on Days 10 and 20 for immunofluorescence staining. Quantitative assessment of marker expression was performed in ImageJ using the mean fluorescence intensity function to ensure robust statistical comparison.

### 2.6 Characterization of beige adipocytes

Real-time PCR was performed as described above to assess adipogenic, thermogenic, beige-associated and m6A regulatory genes at the indicated differentiation stages. Primer sequences are provided in Supplementary Table S1. On days 10 and 20 of differentiation, cells were fixed to be subjected to immunostaining, following the immunocytochemistry protocol described in Section 2.4, to investigate the expression of beige adipogenesis markers. Adipose progenitor markers PDGFRα, CD44, and CD29 were visualized on day 10 of differentiation. Adipogenic regulatory markers CEBPA and PPARG, and thermogenic adipocyte markers CITED1, UCP1, and PRDM16 were also examined on day 20 of adipogenesis. Cells were examined and imaged at 20x using an Olympus Microscope IX73. A minimum of 4 images per fixed well was considered when analyzing using the mean fluorescent intensity function in ImageJ to achieve better statistics.

On the final day (day 20) of differentiation, live cells were incubated with 1 μmol/L MitoTracker Red CMXRos (M7512, Invitrogen) diluted in phosphate buffer saline (PBS) for 45 min at 37 °C in a humidified incubator with 5% CO_2_. After staining, cells were fixed with 4% paraformaldehyde for 10 min at room temperature, then washed twice with PBS. Nuclei were counterstained with DAPI, and cells were subsequently imaged using Olympus Microscope IX73. On the 20th day of differentiation, cells were fixed with 4% paraformaldehyde (PFA) for 10 min at room temperature, then rinsed three times with cold PBS. Subsequently, the cells were incubated with HCS LipidTOX Green Neutral Lipid Stain (Invitrogen) diluted 1:200 in buffer (PBS) for 45 min at room temperature. After a 5-min incubation with DAPI, the cells were visualized using Olympus Microscope IX73. Cells differentiated for 20 days were fixed with 4% paraformaldehyde for 10 min at room temperature and washed three times with ice-cold PBS. Following fixation, cells were incubated with 100% propylene glycol for 2 min at room temperature, then stained with Oil Red O solution (Sigma, O1516) for 15 min, following the manufacturer’s protocol. After staining, cells were finally maintained in PBS.

### 2.7 m6A analysis using RNA dot blot

To assess global m6A RNA levels, total RNA was spotted (500 and 1000 ng per sample) onto a nylon membrane. After air-drying (5 min), the RNA was UV crosslinked at 120 mJ/cm2 (254 nm) using a Spectrolinker XL-1500. The membrane was then washed with 0.1% Phosphate buffer saline-Tween (PBST), blocked with 5% skimmed milk in PBST for 1 h, and incubated overnight at 4 °C with an anti-m6A primary antibody (Abcam, ab151230, 1:1,000) diluted in blocking buffer. After washing, the membrane was incubated for 1 h with an HRP-conjugated goat anti-rabbit IgG secondary antibody (Invitrogen, 1:2,500), followed by additional washes. Detection was performed using ECL reagents (ThermoFisher) and chemiluminescence imaging (Biorad ChemiDoc) (Dong et al., 2023; Zhao et al., 2025). To assess RNA loading, the membrane was stained with 0.1% methylene blue, and the m6A signal intensity was normalized to the corresponding methylene-blue signal. The dot intensity was quantified using Image Lab software (Biorad). Three independent dot-blot experiments were performed. The normalized values were expressed relative to the corresponding control.

## 3 Results and discussions

### 3.1 Generation and characterization of HDF-derived iPSC lines

Adult (HDFa) and neonatal (HDFn) human dermal fibroblasts were successfully reprogrammed using Sendai virus-based reprogramming. Colonies with typical iPSC morphology were established and expanded, and clearance of Sendai transgenes was confirmed by RT-PCR. Both HDFa and HDFn-derived iPSC lines expressed the pluripotency markers OCT4, TRA-1-60 and SSEA4 and showed alkaline phosphatase activity. Pluripotency was further supported by increased expression of OCT4, NANOG, SOX2 and REX1 relative to parental fibroblasts and by the ability of both lines to differentiate toward ectodermal, mesodermal and endodermal lineages (Supplementary Figures S1, S2). These characterized iPSC lines were subsequently used to investigate the effect of transient METTL3 inhibition during beige adipogenesis.

The HDFa and HDFn-derived iPSC lines were selected for an *in vitro* investigation of beige adipogenesis, highlighting the importance of METTL3 and its role in m6A during this process. Comparisons between HDFa- and HDFn-derived iPSC lines are exploratory and descriptive; each donor origin is represented by a single independently derived iPSC line.

### 3.2 Early METTL3 inhibition with STM2457 alters m6A levels and beige adipogenic differentiation

#### 3.2.1 Response of HDFa-iPSCs to STM2457

To examine the contribution of METTL3-dependent m6A regulation during early adipogenic commitment, HDFa-iPSCs were treated with STM2457 from day 0 to day 4 and subsequently differentiated until day 20 (Figure 2A). By Day 10, cells showed loss of compact iPSC morphology, indicating transition into the adipogenic lineage. Samples were evaluated at defined stages of differentiation to assess global m6A abundance, gene expression and adipogenic phenotypes. Global m6A levels were assessed during differentiation by RNA dot blot analysis (Figure 2B). STM2457 treatment reduced m6A abundance during the early treatment period compared with the vehicle control. This may indicate an effective inhibition of m6A deposition during early adipogenesis. Following inhibitor withdrawal, m6A levels showed partial recovery at later differentiation stages but remained altered relative to the corresponding controls, likely due to the withdrawal of the METTL3 inhibitor. Together, these observations indicate that transient STM2457 exposure during early differentiation produced changes in global m6A abundance that extended beyond the treatment period. Expression of genes involved in m6A regulation was also examined during differentiation (Figure 2D). METTL3 and METTL14 showed relatively modest changes following STM2457 exposure, whereas FTO and ZC3H13 increased at selected later time points. The changes observed after inhibitor withdrawal may reflect cellular adaptation to altered m6A regulation. The transcriptional upregulation could reflect a feedback response to reduced enzymatic activity, as STM2457 competitively inhibits METTL3 without blocking transcription (Yankova et al., 2021). WTAP and ALKBH5 remained comparatively stable. These stage-dependent transcriptional changes were accompanied by transient inhibition of METTL3, which was associated with alterations in the expression of components of the m6A regulatory machinery.

**FIGURE 3:**
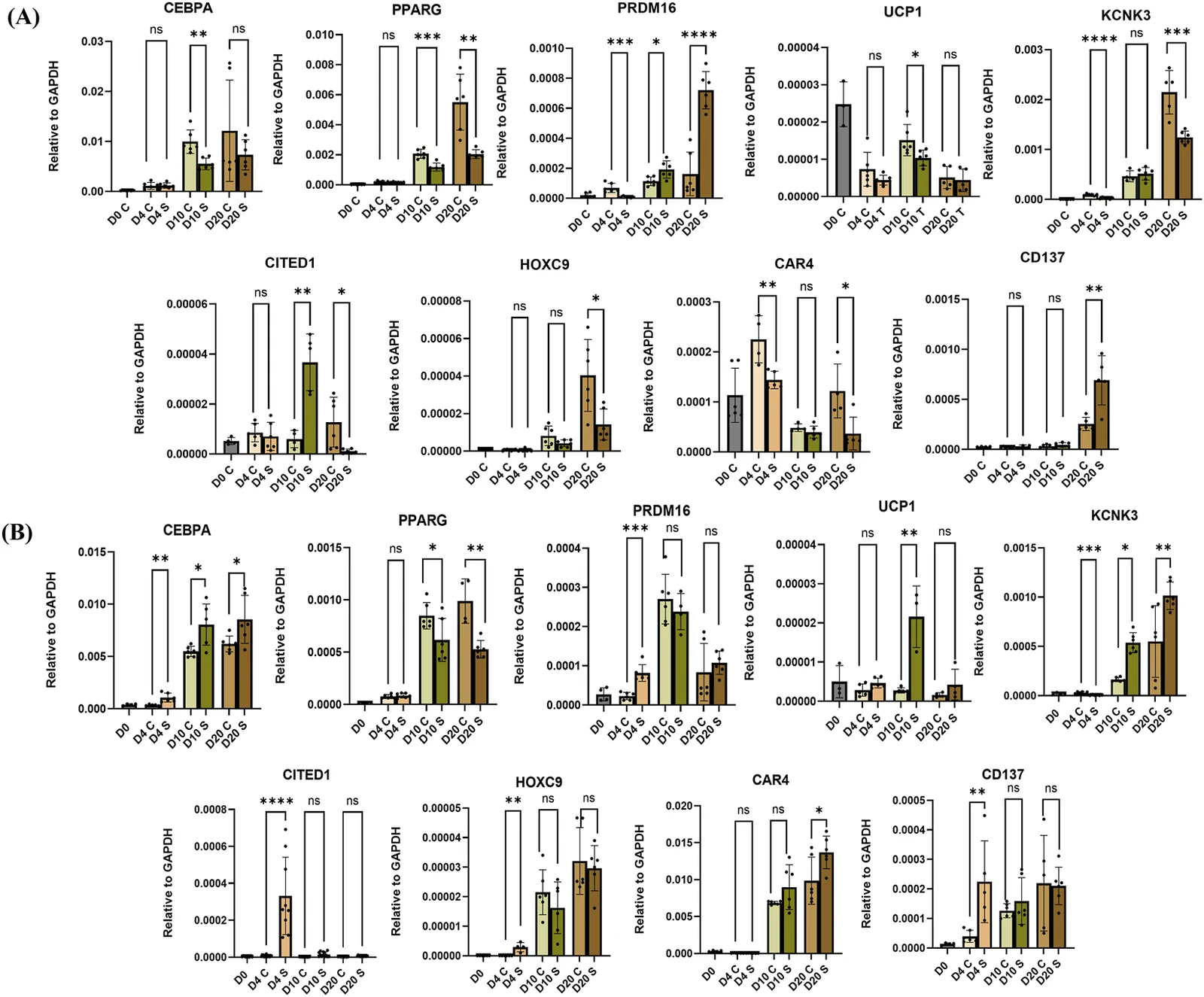
Time-course expression of adipogenic, thermogenic and beige-associated genes during STM2457-treated beige adipogenesis. **(A)** HDFa-iPSCs and **(B)** HDFn-iPSCs. C, control; S, STM2457. Gene expression was normalized to GAPDH. Individual data points show technical duplicate measurements from three independent wells per condition. Control and STM2457-treated groups were compared at each time point using a two-tailed unpaired t-test. Data are presented as mean ± SD. Statistical significance is indicated as *p < 0.05, **p < 0.01, ***p < 0.001, ****p < 0.0001; ns, not significant.

At the progenitor stage, expression of CD29, CD44, and PDGFRA was evaluated by immunofluorescence (Supplementary Figure S3A). CD29 and PDGFRA showed relatively small differences between control and STM2457-treated cells, whereas CD44 fluorescence was reduced following STM2457 treatment. We next examined whether these early changes were accompanied by altered expression of adipogenic and thermogenic markers during subsequent differentiation (Figure 3A).

In comparison with the control group, STM2457-treated cells exhibited a blunted induction of the core adipogenic transcription factors CEBPA and PPARG at several stages of differentiation (Figure 3A). Immunofluorescence analysis at day 20 similarly showed lower CEBPA and PPARG signals in STM2457-treated cells (Supplementary Figure S4). In controls, PRDM16, UCP1, CITED1, and beige specific markers, HOXC9, KCNK3, CAR4, and, CD137, increased progressively during differentiation (Figure 3A). Early exposure to STM2457 significantly attenuated the induction of most genes by day 20. Early METTL3 inhibition appears to disrupt the establishment of the thermogenic program broadly.

CD137 expression increased by day 20, aligning with diminished thermogenic marker expression and this may indicate altered beige adipocyte maturation (Srivastava et al., 2020). PRDM16, being a key beige adipocyte regulator, exhibited a biphasic pattern. It was affected at the early stage; however, substantial upregulation was observed by day 20. Individual components of the beige-associated transcriptional program may respond differently following transient METTL3 inhibition. The late increase in PRDM16 may represent a compensatory transcriptional response that contributes to partial restoration of the beige-associated program; however, this interpretation requires further validation. The IF analysis showed limited differences in UCP1 and PRDM16 protein levels at the mature stage, as shown in Supplementary Figure S4.

**FIGURE 4:**
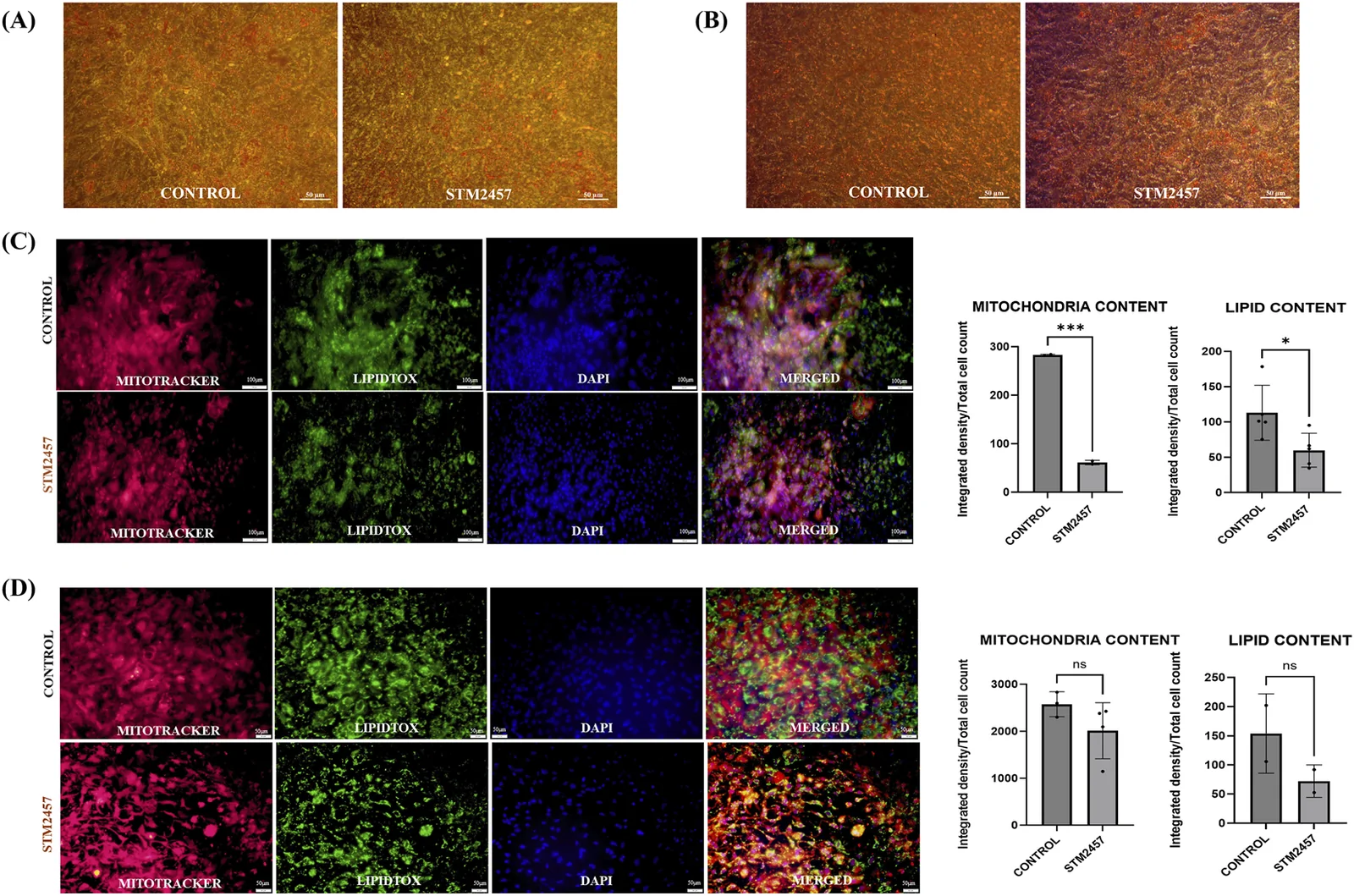
Phenotypic assessment of STM2457-treated cells on day 20 of beige adipogenesis. Representative Oil Red O images acquired at ×20 magnification are shown for **(A)** HDFa-derived and **(B)** HDFn-derived cells under control and STM2457-treated conditions; scale bar: 50 µm. Representative MitoTracker Red, LipidTOX, DAPI and merged fluorescence images with corresponding quantitative analysis are shown for **(C)** HDFa-iPSCs and **(D)** HDFn-iPSCs; scale bar: 100 µm. Fluorescence intensity was quantified as integrated density normalized to total cell count across ≥4 fields of view from a single well per condition from a single iPSC line within one experiment. Individual data points represent fields of view; outliers were removed. Statistical analysis was performed on averaged values per group using a two-tailed unpaired t-test. Significance is indicated as *p < 0.05, **p < 0.01, ***p < 0.001; ns, not significant.

Oil Red O staining at day 20 qualitatively suggested lower lipid accumulation in STM2457-treated HDFa-derived cells. Both whole-well staining and representative magnified (×20) images are provided to show the overall staining pattern and cellular morphology, respectively (Figures 2C, 4A). Consistent with the Oil Red O staining, LipidTOX fluorescence was lower and MitoTracker fluorescence was reduced in STM2457-treated cultures (Figure 4C). Overall, transient STM2457 treatment during the early differentiation window was associated with persistent changes in adipogenic and thermogenic markers and with reduced lipid and mitochondrial staining in the HDFa-derived iPSC line.

#### 3.2.2 Response of HDFn-iPSCs to STM2457

The effect of transient STM2457 treatment was examined in HDFn-iPSCs using the same differentiation protocol. Global m6A abundance was reduced following STM2457 exposure and remained lower than the corresponding controls at later stages of differentiation (Figure 2E). The persistence of reduced m6A differed from pattern observed in the HDFa-derived line, in which a greater degree of recovery was observed after inhibitor withdrawal. Expression of m6A regulatory genes also differed following STM2457 treatment (Figure 2G). METTL3 expression increased mainly at the later stage, while METTL14, FTO and ALKBH5 showed stage-dependent increases during differentiation.

This delayed increase in METTL3 expression could be a late-stage compensatory attempt. METTL14, FTO, and ALKBH5 expressions increased during adipogenesis, suggesting an attempt to rebalance m6A dynamics in the absence of early METTL3 activity. These interpretations remain preliminary and require further studies to confirm the underlying mechanisms. Despite these transcriptional changes, global m6A levels remained reduced relative to controls. Thus, changes in expression of the measured m6A regulators were not accompanied by complete restoration of global m6A abundance during the experimental period.

Despite early m6A loss, HDFn-iPSCs were able to initiate adipogenesis. On day10, IF analysis showed stable adipogenic induction markers, CD29 and CD44; however, PDGFRA protein expression appeared to decrease (Supplementary Figure S3B). The later adipogenic and thermogenic transcriptional response was subsequently evaluated by RT-PCR (Figure 3B). STM2457-treated HDFn-iPSCs showed lower PPARG expression at later stages, whereas CEBPA remained comparatively elevated at the transcript level. On day 20, however, IF signals for both adipogenic regulators were reduced in STM2457-treated cultures (Supplementary Figure S5), indicating that the transcriptional and protein-level responses were not fully concordant. Several beige and thermogenic-associated transcripts showed increased expression at selected stages following STM2457 treatment. PRDM16, HOXC9 and CITED1 increased at earlier stages, whereas UCP1, CAR4 and KCNK3 were elevated at later time points (Figure 3B). Despite these transcriptional changes, CITED1, PRDM16 and UCP1 IF signals showed limited difference on day 20 (Supplementary Figure S5). Oil Red O staining suggested reduced lipid accumulation in STM2457-treated HDFn-derived cells at day 20 (Figures 2F, 4B). LipidTOX and MitoTracker fluorescence also tended to be lower, although the differences were not statistically significant in the analyzed fields (Figure 4D).

#### 3.2.3 Comparison of STM2457 responses between the two iPSC lines

Early METTL3 inhibition by STM2457 revealed distinct responses between the two iPSC lines during adipogenesis. In HDFa-iPSCs, the METTL3 inhibition led to persistent defects in adipogenic and thermogenic differentiation, with incomplete recovery of m6A levels and impaired lipid and mitochondrial maturation at later stages. On the other hand, HDFn-iPSCs exhibited better transcriptional plasticity under similar conditions. HDFn-iPSCs exhibited a different response profile during early stages of differentiation. Partial activation of adipogenic and thermogenic processes was undertaken despite sustained m6A reduction. Therefore, early METTL3 activity was associated with differentiation outcomes in both lines, with more pronounced effects observed in HDFa-iPSCs. HDFn-iPSCs appear to retain greater capacity to adapt, even though thermogenic-associated phenotype was compromised. These observations indicate line-specific responses to transient METTL3 inhibition.

### 3.3 Early METTL3 inhibition with UZH2 produces cell-line dependent responses

#### 3.3.1 Response of HDFa-iPSCs to UZH2

**FIGURE 5:**
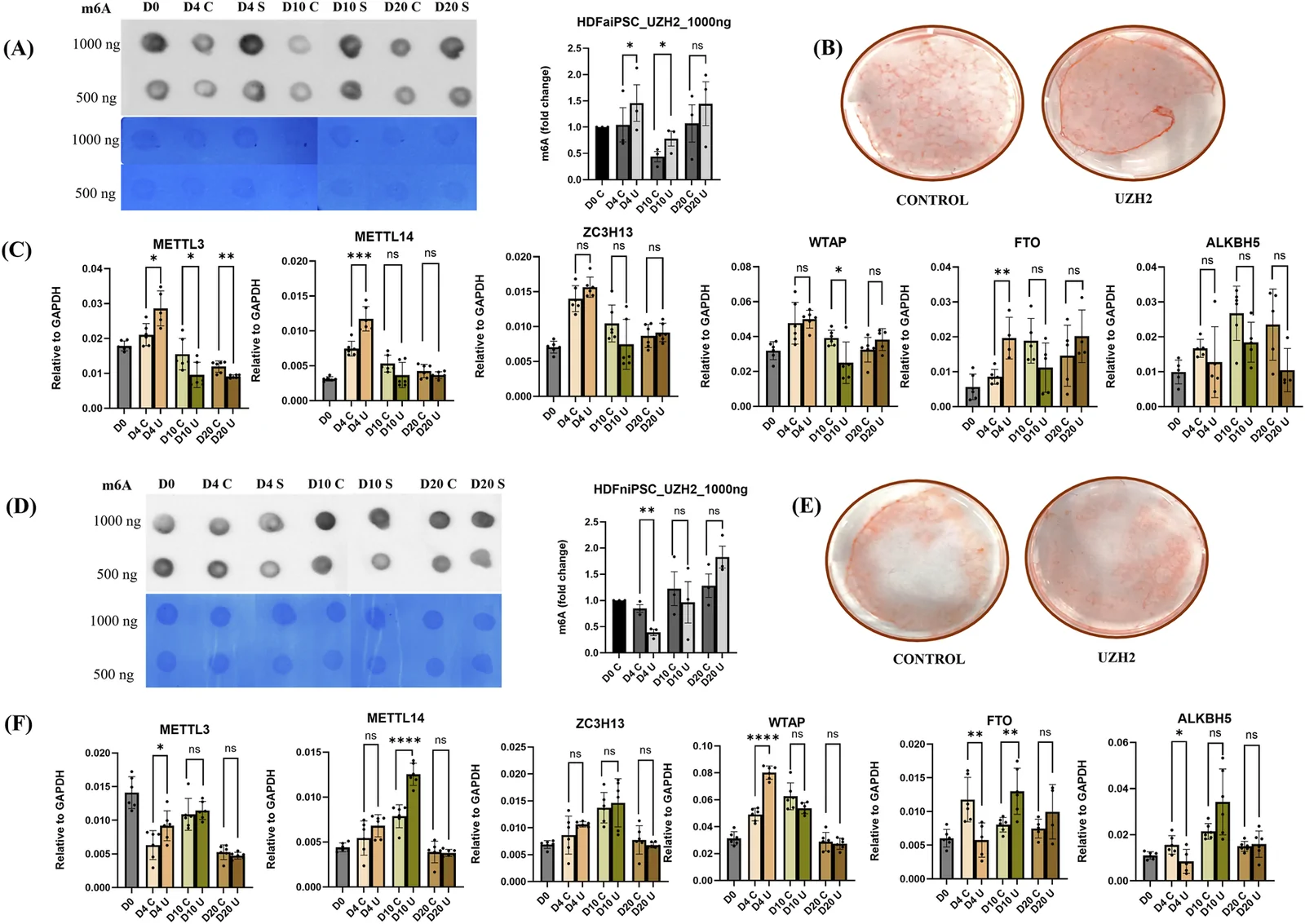
Effects of transient UZH2 treatment during beige adipogenesis. **(A)** Representative m6A RNA dot blot and corresponding methylene-blue loading control for HDFa-iPSCs, with quantification of normalized m6A abundance. **(B)** Whole-well Oil Red O staining of control and UZH2-treated HDFa-derived cells at day 20. **(C)** Time-course expression of m6A regulatory genes in HDFa-iPSCs. **(D)** Representative m6A RNA dot blot and corresponding methylene-blue loading control for HDFn-iPSCs, with quantification of normalized m6A abundance. **(E)** Whole-well Oil Red O staining of control and UZH2-treated HDFn-derived cells at day 20. **(F)** Time-course expression of m6A regulatory genes in HDFn-iPSCs. C, control; U, UZH2. For m6A analysis, data represent mean ± SEM from three independent dot-blot experiments after normalization to the corresponding methylene-blue signal; quantification shown corresponds to the 1000-ng RNA spots. Matched control and UZH2-treated samples from the independent experiments were compared at each time point using a paired t-test. For qPCR **(C,F)**, individual points show technical duplicate measurements from three independent wells per condition. Control and UZH2-treated groups were compared at each time point using a two-tailed unpaired t-test. Data are presented as mean ± SD. Statistical significance is indicated as *p < 0.05, **p < 0.01, ***p < 0.001, ****p < 0.0001; ns, not significant.

HDFa-iPSCs were treated with UZH2 from day 0 to day 4 and differentiated using the same protocol applied to STM2457 experiments. By day 10, the cells began to lose their compact iPSC morphology, as expected. This was consistent with the adipogenic commitment. It is to be noted that small clusters or clump-like structures were observed during the later stages of differentiation. Global m6A abundance was assessed across differentiation by RNA dot blot analysis (Figure 5A). m6A levels were higher in UZH2-treated HDFa-iPSCs in comparison to the corresponding controls, especially at days 4 and 10. This pattern differed from the reduction observed following STM2457 treatment. This increase was detectable at early stages of differentiation and remained sustained at later timepoints. These findings may suggest incomplete or off-target inhibition, potentially involving other methyltransferases, or compensatory feedback upregulating methylation. Prior literature indicates UZH2’s cell-type-dependent efficacy and partial target non-specificity (Dolbois et al., 2021). Therefore, this can be considered an anomalous pharmacological observation, and the mechanism underlying the increase in m6A is yet to be resolved. The short treatment window may have allowed METTL3 activity to recover, contributing to m6A elevation. A persistent rise in global m6A during differentiation may alter the stability of transcripts required for thermogenic lineage specification, thereby skewing results. Transcriptional analysis of m6A regulatory components revealed moderate, stage-dependent changes following METTL3 inhibition.

Expression of m6A regulatory genes showed stage-dependent changes following UZH2 treatment (Figure 5C). METTL3, METTL14 and FTO were elevated at selected early time points, whereas METTL3 expression was lower than control at later stages. WTAP and ZC3H13 showed comparatively limited changes. These transcriptional responses indicate that UZH2 exposure was accompanied by alterations in the expression of several components of the m6A regulatory machinery, although their relationship to the unexpected global m6A pattern remains unclear.

**FIGURE 6:**
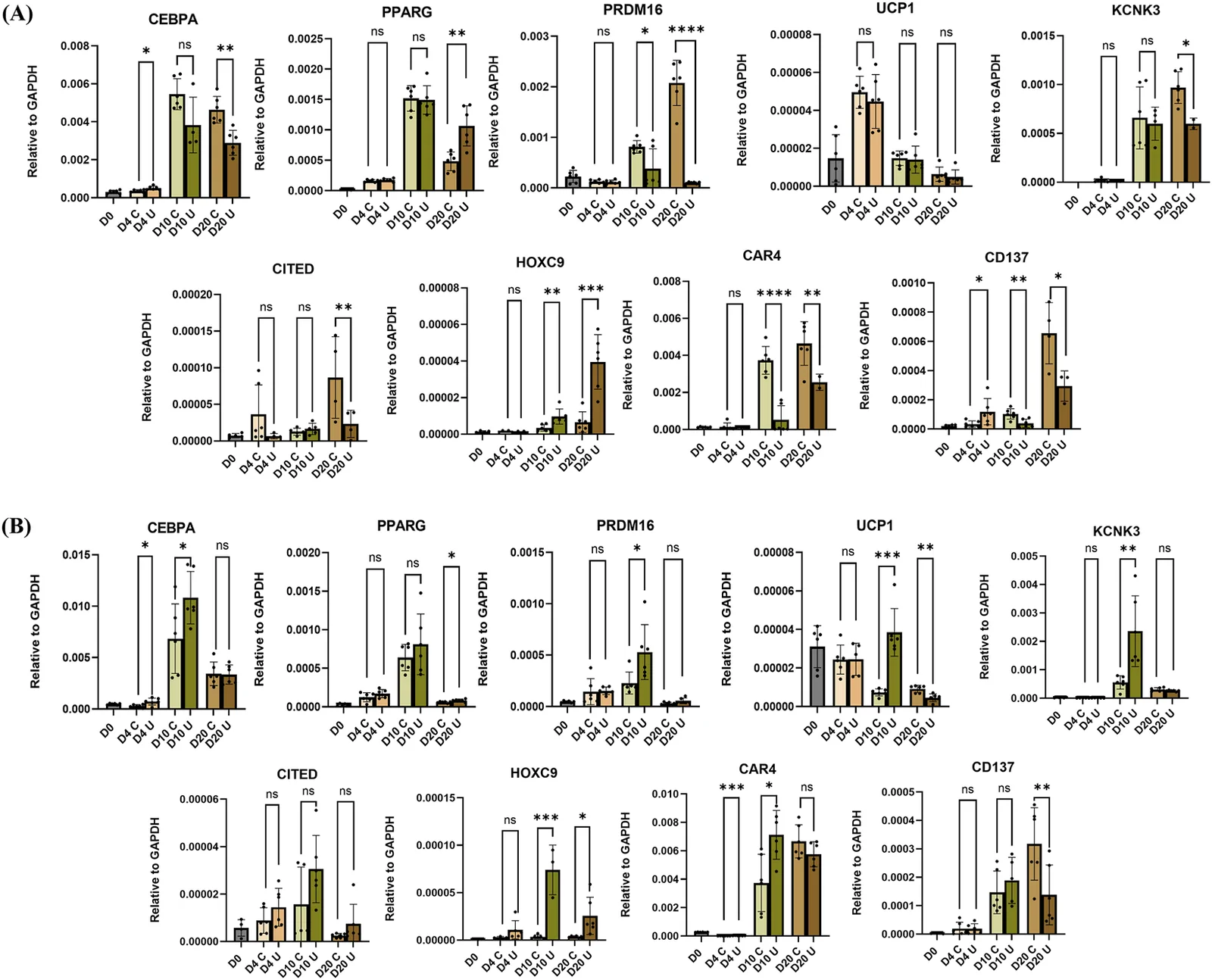
Time-course expression of adipogenic, thermogenic and beige-associated genes during UZH2-treated beige adipogenesis. **(A)** HDFa-iPSCs and **(B)** HDFn-iPSCs. C, control; U, UZH2. Gene expression was normalized to GAPDH. Individual data points show technical duplicate measurements from three independent wells per condition. Control and UZH2-treated groups were compared at each time point using a two-tailed unpaired t-test. Data are presented as mean ± SD. Statistical significance is indicated as *p < 0.05, **p < 0.01, ***p < 0.001, ****p < 0.0001; ns, not significant.

At the progenitor stage, UZH2-treated HDFa-iPSCs showed higher CD29 and PDGFRA IF signals, whereas CD44 showed comparatively little change (Supplementary Figure S6A). As shown in Figure 6A, CEBPA was upregulated by day 4 and declined by day 20, whereas PPARG increased significantly by day 20. This reflects an initial priming of adipogenesis followed by recovery of terminal differentiation, even after METTL3 inhibition. However, PRDM16 was significantly downregulated on days 10 and 20, suggesting a loss of beige adipocyte identity. The reduced expression of UCP1, KCNK3, CITED1, CAR4 and CD137 supported this. There was a concurrent increase in HOXC9, which is typically higher in white adipocytes. These expression changes were consistent with reduced maintenance of the beige-associated transcriptional program. Therefore, these findings could be consistent with a whitening-like shift. The IF analysis was in line with these findings (Supplementary Figure S7). A decrease in UCP1 and an increase in PPARG at the protein level by day 20 suggest impaired beige adipocyte maturation and may indicate a shift from beige adipogenesis towards whitening.

**FIGURE 7:**
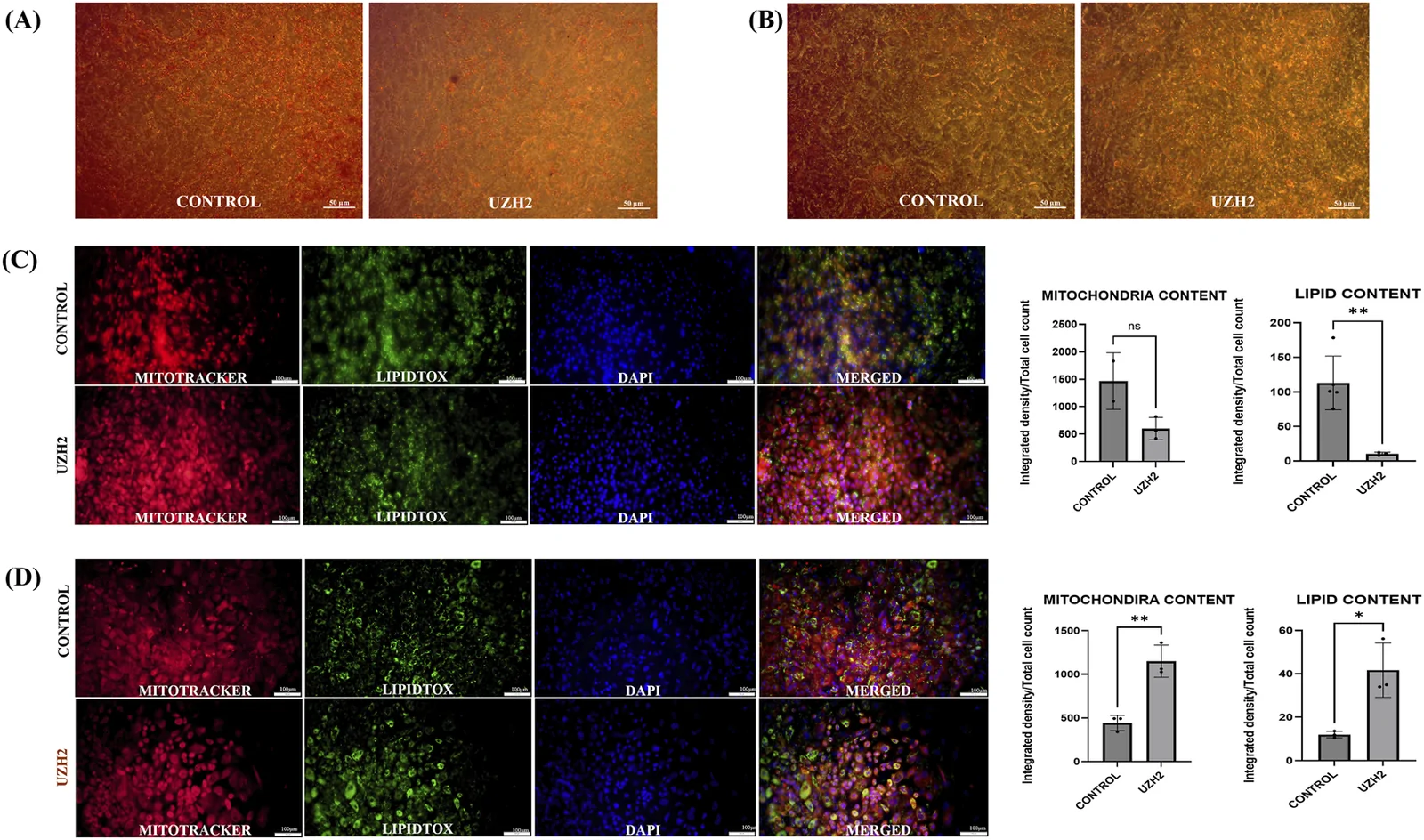
Phenotypic assessment of UZH2-treated cells on day 20 of beige adipogenesis. Representative Oil Red O images acquired at ×20 magnification are shown for **(A)** HDFa-derived and **(B)** HDFn-derived cells under control and UZH2-treated conditions; scale bar: 50 µm. Representative MitoTracker Red, LipidTOX, DAPI and merged fluorescence images with corresponding quantitative analysis are shown for **(C)** HDFa-iPSCs and **(D)** HDFn-iPSCs; scale bar: 100 µm. Fluorescence intensity was quantified as integrated density normalized to total cell count across ≥4 fields of view from a single well per condition from a single iPSC line within one experiment. Individual data points represent fields of view; outliers were removed. Statistical analysis was performed on averaged values per group using a two-tailed unpaired t-test. Significance is indicated as *p < 0.05, **p < 0.01, ***p < 0.001; ns, not significant.

Oil Red O staining qualitatively showed reduced lipid accumulation in UZH2-treated HDFa-derived cells at day 20 (Figures 5B, 7A), while LipidTOX quantification showed significantly lower neutral-lipid staining (Figure 7C). This pattern is consistent with previous studies highlighting lipid loss during adipocyte whitening. Beige adipocyte whitening has been described as a multistep process involving early metabolic regression and lipid remodeling prior to the acquisition of white-like characteristics (Altshuler-Keylin et al., 2016; Rosen and Spiegelman, 2014). This further raises the possibility of a drift away from the beige thermogenic program toward a whitening-like adipocyte state, although terminal differentiation is impaired.

#### 3.3.2 Response of HDFn-iPSCs to UZH2

The response to transient UZH2 treatment was next examined in HDFn-iPSCs. Global m6A abundance decreased during the early treatment period and remained reduced at day 10 relative to controls (Figure 5D). This represented a clearer early reduction in global m6A than that observed in HDFa-derived line. At the later stage, m6A levels approached or exceeded control values, indicating a different temporal response from that observed during the inhibitor-exposure period. UZH2 treatment was also associated with stage-dependent changes in m6A regulatory gene expression (Figure 5F). METTL3 and WTAP increased during the early treatment period, while METTL14 showed a later increase. Cells sense reduced m6A-dependent processes and upregulate writer components to restore methylation homeostasis (Yankova et al., 2021). FTO and ALKBH5 were reduced during early differentiation and increased following inhibitor withdrawal. These data demonstrate a dynamic transcriptional response of the m6A regulatory machinery during and after UZH2 exposure.

At the progenitor stage, UZH2-treated HDFn-iPSCs showed altered expression of the surface markers examined, with the most evident change observed for CD29 (Supplementary Figure S6B). These changes may reflect a transient delay in commitment rather than complete inhibition of adipogenic differentiation. During subsequent differentiation, CEBPA and PPARG were elevated at selected time points in UZH2-treated cells (Figure 6B). Several thermogenic and beige-associated genes, including PRDM16, UCP1, CITED1, CAR4, HOXC9 and KCNK3, also showed transient increases, particularly around the intermediate stage of differentiation.

IF at day 20 showed increased adipogenic-marker signals in the UZH2-treated cultures (Supplementary Figure S8). Oil Red O staining qualitatively showed greater lipid accumulation in UZH2-treated HDFn-derived cells at day 20 (Figures 5E, 7B). LipidTOX and MitoTracker fluorescence were also increased in the analyzed fields (Figure 7D). Thus, despite transient early changes in global m6A abundance, this iPSC line retained the ability to progress toward an adipocyte phenotype following UZH2 withdrawal.

#### 3.3.3 Comparison of UZH2 responses between the two iPSC lines

The two iPSC lines showed different responses to transient UZH2 exposure. In the HDFa-derived line, treatment was associated with an unexpected global m6A profile, reduced expression of several beige-associated markers and lower lipid accumulation on day 20. In the HDFn-derived line, early m6A reduction was followed by later recovery together with preservation of adipogenic differentiation and increased lipid and MitoTracker staining. These differences indicate that the cellular response to UZH2 was strongly dependent on the iPSC lines examined.

### 3.4 Comparison of the responses to STM2457 and UZH2

STM2457 and UZH2 produced distinct responses during early beige adipogenesis. STM2457 was associated with reduced global m6A abundance in both iPSC lines during the early treatment period, whereas the response to UZH2 was more variable between the two lines. The downstream transcriptional and phenotypic responses also differed according to both the inhibitor and the iPSC line. These findings indicate that the biological consequence observed following pharmacological METTL3 inhibition cannot be attributed solely to a uniform reduction in global m6A and should therefore be interpreted in the context of the specific inhibitor and cellular model used. The observed differences between HDFa and HDFn-derived iPSCs may reflect variability in cellular response to METTL3 inhibition (Polo et al., 2010). Multiple factors, including donor origin, reprogramming-associated variability, or clonal differences, may influence sensitivity to early disruptions in m6A signaling (Bar-Nur et al., 2015; Kim et al., 2010). In contrast, the neonatal-derived iPSC line demonstrated greater recovery capacity following transient epitranscriptomic disruption, consistent with enhanced adaptive response dynamics (Lin et al., 2024; Ohi et al., 2011).

## 4 Conclusion

This study systematically explores the role of METTL3-dependent m6A regulation in early beige adipogenesis using human iPSCs derived from adult and neonatal human dermal fibroblasts, and two chemically distinct METTL3 inhibitors, STM2457 and UZH2. This work provides insight into how epitranscriptomic control influences adipocyte lineage commitment, maturation, and thermogenic capacity by integrating time-course m6A profiling, transcriptional and protein-level studies, and phenotypic indicators of metabolic maturation. Across both cell lines, the early differentiation timeline (days 0–4) emerged as a critical and sensitive period during which transient METTL3 inhibition exerted uneven influence downstream. STM2457, a selective METTL3 inhibitor, consistently reduced global m6A levels during this time frame and produced predictable, stage-dependent consequences that persisted beyond inhibitor withdrawal. Among HDFa-derived iPSCs, perturbation of this early window resulted in sustained impairment of adipogenic and thermogenic marker expression, reduced lipid accumulation, and diminished MitoTracker fluorescence at later stages, indicating compromised differentiation. However, HDFn-iPSCs exhibited greater transcriptional plasticity, with partial recovery of adipogenic and thermogenic marker expression following early inhibition, although phenotypic markers of metabolic maturation remained lower relative to non-treated controls.

On the other hand, UZH2 yielded inconsistent outcomes that varied by cell type. In HDFa-iPSCs, early treatment increased global m6A levels and suppressed beige-specific molecular traits. These findings suggest that UZH2-induced disruption of the m6A pathway occurs early in differentiation, ultimately impairing the beige phenotype. In HDFn-iPSCs, UZH2 treatment more effectively lowered the m6A levels during early differentiation and was accompanied by coordinated induction of adipogenic and beige-associated markers. This was followed by increased lipid storage and mitochondrial staining intensity at later stages, suggesting that the cells achieved enhanced phenotypic maturation after recovering from the initial m6A modulation.

Collectively, the findings support a temporal role for METTL3-mediated m6A regulation during early adipogenic commitment. Perturbation of METTL3 activity during this early window was associated with downstream changes that persisted after inhibitor withdrawal. Its activity during the early commitment phase appears vital for the correct sequencing of differentiation. This research underscores the context-dependent role of METTL3-mediated m6A regulation during beige adipogenesis, as two human iPSC lines of distinct donor origin exhibited differential transcriptional responses to transient METTL3 inhibition, suggesting that cellular context modulates epitranscriptomic sensitivity during early lineage commitment. Whether this reflects donor age, epigenetic memory, or clonal variation warrants investigation in larger, multi-line cohorts. By systematically comparing adult and neonatal fibroblast-derived iPSCs and employing chemically distinct METTL3 inhibitors, this study reveals that m6A may not operate as a uniform regulator of adipocyte fate, but rather modulates differentiation robustness, lineage fidelity, and adaptive capacity in a cell line-specific manner. These findings provide an important conceptual framework for interpreting m6A-dependent phenotypes in human stem cell models and highlight the need to consider cellular origin and epigenetic context when targeting epitranscriptomic pathways in adipose biology and related translational applications.

## 5 Limitations and future directions

This study focuses on the role of METTL3-mediated m6A regulation during the early commitment phase of beige adipogenesis and establishes a human iPSC-based framework to examine the epitranscriptomic control of differentiation. The observed differences may reflect line-specific variation shaped by donor origin, clonal effects, and reprogramming, and motivate validation across additional iPSC lines. While METTL3 inhibition was intentionally confined to the early window, extending to later stages may further clarify stage-specific m6A requirements during adipocyte maturation. Lipid accumulation and MitoTracker fluorescence were assessed as phenotypic indicators of metabolic maturation. Incorporation of direct mitochondrial functional analysis would complement these findings by linking m6A regulation to thermogenic activity. In addition, transcriptome-wide approaches such as m6A sequencing and RNA sequencing could expand insight into transcript-specific regulatory mechanisms. Finally, adapting this framework to brown adipogenesis would help distinguish shared and lineage-specific epitranscriptomic programs governing thermogenic fat development.

## Data Availability

The original contributions presented in the study are included in the article/Supplementary Material, further inquiries can be directed to the corresponding author.
