# Supplementary material for: Impact of METTL3-mediated m6A regulation on early human beige adipogenesis from hiPSCs
Source: Front Cell Dev Biol. 2026 Sep 11;14:1918478. doi: 10.3389/fcell.2026.1918478 (PMC13612436; doi:10.3389/fcell.2026.1918478)
Supplement: Supplementary file 1 [file DataSheet1.pdf]

1. Supplementary Tables and Figures

Supplementary Table 1: Primer sequences for Sendai Transgenes, pluripotency markers, m6A regulator markers, thermogenic markers and beige adipocyte marker genes

Target gene	Forward/Reverse (5'-3')	Target gene	Forward/Reverse (5'-3')
SeV (181 bp)	GGATCACTAGGTGATATCGAGC<br>ACCAGACAAGAGTTTAAGAGATATGTATC	HOXC9	GCAGCAAGCACAAAGAGGA<br>CGTCTGGTACTTGGTGTAGGG
KOS (528 bp)	ATGCACCGCTACGACGTGAGCGC<br>ACCTTGACAATCCTGATGTGG	KCNK3	CCTTCTACTTCGCCATCACC<br>GAACATGCAGAACACCTTGC
Klf4 (410 bp)	TTCCTGCATGCCAGAGGAGCCC<br>AATGTATCGAAGGTGCTCAA	CITED1	ACCGGACTTGGAGTCAGAGA<br>CAGTTTCGCCACCTGAAAAC
c-Myc (532 bp)	TAACTGACTAGCAGGCTTGTCG<br>TCCACATACAGTCCTGGATGATGATG	CD137	AGCTGTTACAACATAGTAGCCAC<br>TCCTGCAATGATCTTGTCCTCT
OCT4	GAGAACCGAGTGAGAGGCAACC<br>CATAGTCGCTGCTTGATCGCTTG	CAR4	TGTCCAAAATAACGGGCACT<br>CAGTCCTCCTCCAGAAATGC
NANOG	ACTATTGAGGTAAAGGGTTAAGCTGT<br>CACAACCAACAAATTAGGGGATA	UCP1	CTCACCGCAGGGAAAGAA<br>GGTTGCCCAATGAATACTGC
SOX2	TTGCTGCCTCTTTAAGACTAGGA<br>TAAGCCTGGGGCTCAAACT	METTL3	CGTACTACAGGATGATGGCTTTC<br>TTTCATCTACCCGTTCATACCC
REX1	TTTCTCCTTTGTTTTACGTTTGG<br>GGCTCCAGCCTCAGTACATT	METTL14	TCAGGCTAAAGGATGAGTTAATAGC<br>CAAAGGCTTCTATATCGGCTTG
GADPH	GAGTCCACTGGCGTCTTCAC<br>TTCACACCCATGACGAACAT	ALKBH5	CAAGCCTATTCGGGTGTCGG<br>GACTTTGTTTCCAACCGGGG
CEBPα	CTTGGGGCTTGGAACCTAA<br>GGAGAGGCGTGGAACTAGAG	ZC3H13	GCTGGAGAAGGATACGACCC<br>CCAGGCTCTCGTGGTTCTTT
PPARγ2	GACAGGAAAGACAACAGACAAATC<br>GGGGTGATGTGTTTGAACTTG	WTAP	ACGCAGGGAAAACATCCTTG<br>TGGGCTTGTTCCAGTTTGTC
PRDM16	TGGCTGCTTCTGGACTCA<br>ATATTATTTACAACGTCACCGTCACT	FTO	TCTGACCCCCAAAGATGATG<br>CTCGGAGAATTAGTTTAGGATATTTCA

Supplementary Table 2: Antibodies used for immunocytochemistry

Antibody	Dilution	Brand/catalogue
Mouse anti-OCT4, Clone 3A2A20, Alexa Fluor® 488-conjugated	1:200	Stem Cell Technologies, Cat# 60093AD
Mouse anti-TRA-1-60, Clone TRA-1-60R, Alexa Fluor® 488-conjugated	1:200	Stem Cell Technologies, Cat# 60064AD
Mouse anti-SSEA-4, Clone MC-813-70, PEconjugated	1:200	Stem Cell Technologies, Cat# 60062PE
Endoderm Markers:		
Mouse anti-GATA-4	1:100	Santa Cruz, Cat# sc-25310
Goat anti-SOX17	1:200	R&D, Cat# AF1924
Mesoderm Markers:		
Goat anti-Brachyury	1:100	R&D, Cat# AF2085
Mouse anti-EOMES	1:100	R&D, Cat# MAB6166
Ectoderm Markers:		
Goat anti- SOX1	1:100	R&D, Cat# AF3369
Mouse anti-Nestin	1:100	Abcam, Cat# ab22035
Adipogenesis markers:		
Rabbit Anti-PDGFRα	1:200	Cell Signaling Technology, Cat# 3174
Mouse Anti-Human CD44	1:50	BD Biosciences, Cat# 550988
Mouse Anti-Human CD29	1:50	BD Biosciences, Cat# 555443
Mouse Anti-PPARγ	1:200	Santa Cruz, Cat# sc-7273
Rabbit Anti-C/EBPα	1:100	Santa Cruz 14A4
Mouse Anti- CITED1	1:200	Novus Biologicals, Cat# H00004435-M03
Mouse Anti-UCP1	1:50	R&D Systems, Cat# MAB6158
Rabbit Anti-PRDM16	1:200	Abcam, Cat# ab106410
Donkey anti-Mouse secondary antibody Alexa Fluor Plus 594	1:1000	Thermo Fisher Scientific, Cat# A32744
Goat anti-Mouse secondary antibody Alexa Fluor Plus 488	1:1000	Thermo Fisher Scientific, Cat# A32723
Goat anti-Rabbit secondary antibody Alexa Fluor Plus 488	1:1000	Thermo Fisher Scientific, Cat# A32731
Donkey anti-Rabbit secondary antibody Alexa Fluor Plus 594	1:1000	Thermo Fisher Scientific, Cat# A32754

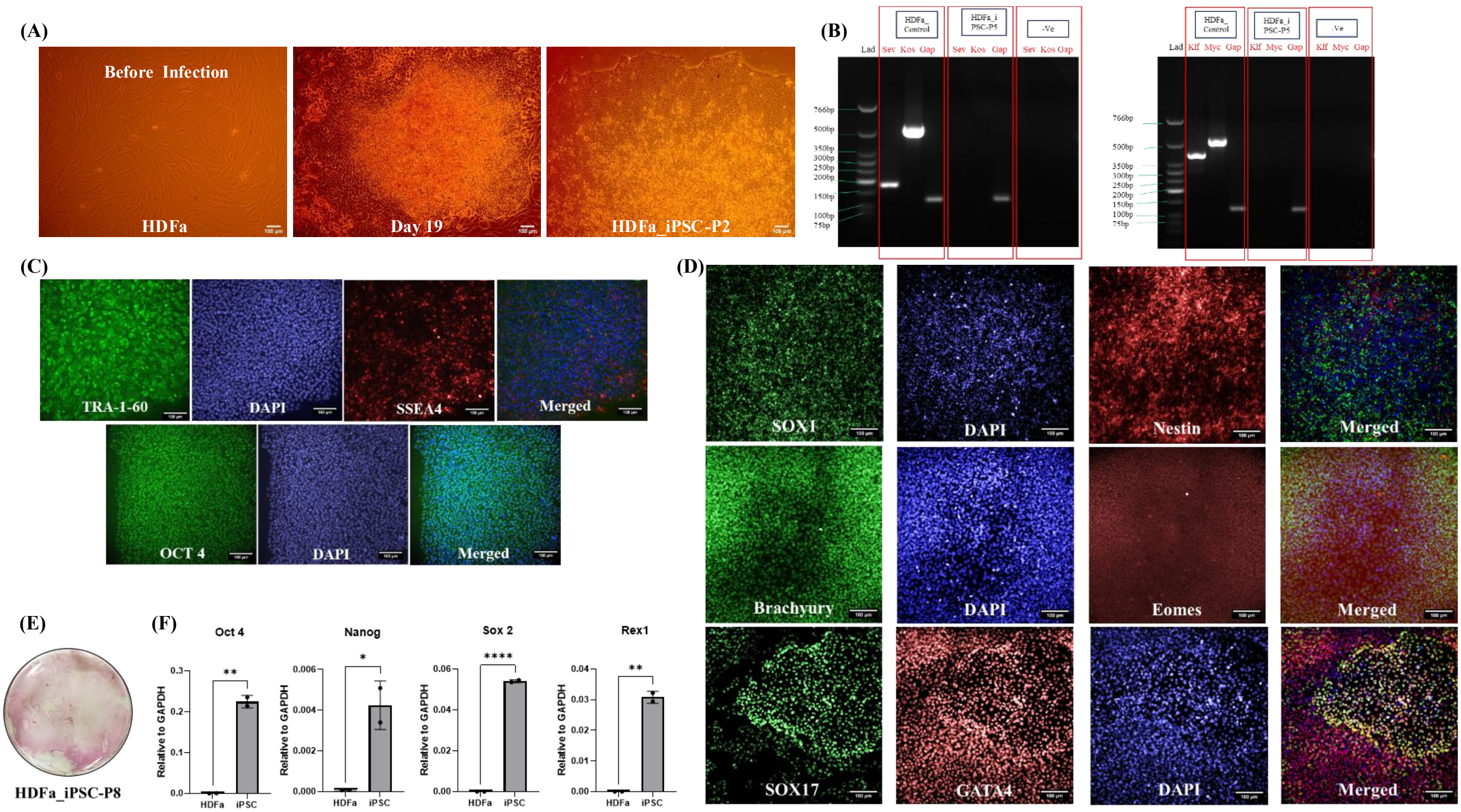

Supplementary Figure S1: (A) Stages of reprogramming HDFa cells upon infection and their development into iPSC colonies; (B) Sendai virus clearance check for HDFa-iPSCs at P5; (C) Immunodetection of pluripotency HDFa-iPSCs; (D) Trilineage differentiation of HDFa-iPSCs with immunofluorescence: Ectoderm specific markers SOX1 and NESTIN, Mesoderm specific markers Brachyury and Eomes, and Endoderm specific markers SOX17 and GATA4, scale bar:100µm; (E) Alkaline phosphatase staining; (F) Gene expression analysis of pluripotency genes from iPSCs generated. RNA collected from single well per condition. Each sample was analyzed in technical duplicate by qPCR which are indicated by data points. Data represents mean ± SD. Statistical analysis was performed on averaged values per group using two-tailed unpaired t-test. Significance indicated as *p<0.05; ns, not significant

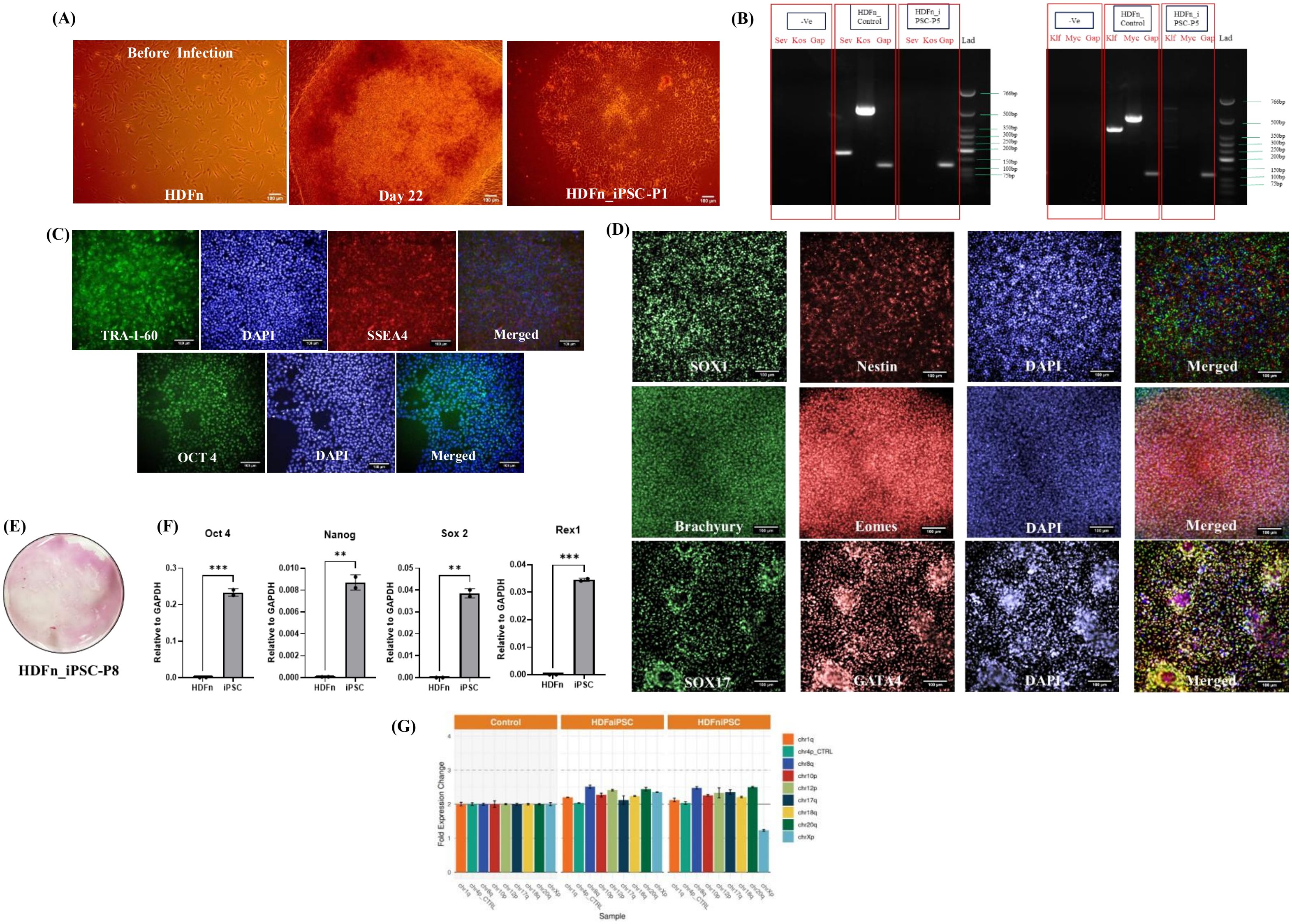

Supplementary Figure S2: (A) Stages of reprogramming HDFn cells upon infection and their development into iPSC colonies; (B) Sendai virus clearance check for HDFn-iPSCs at P5; (C) Immunodetection of pluripotency HDFn-iPSCs; (D) Trilineage differentiation of HDFn-iPSCs with immunofluorescence: Ectoderm specific markers SOX1 and NESTIN, Mesoderm specific markers Brachyury and Eomes, and Endoderm specific markers SOX17 and GATA4, scale bar:100µm; (E) Alkaline phosphatase staining; (F) Gene expression analysis of pluripotency genes from iPSCs generated. RNA collected from single well per condition. Each sample was analyzed in technical duplicate by qPCR which are indicated by data points. Data represents mean ± SD. Statistical analysis was performed on averaged values per group using two-tailed unpaired t-test. Significance indicated as *p<0.05; ns, not significant. (G) Stem cell karyotype results for HDFa-iPSCs and HDFn-iPSCs

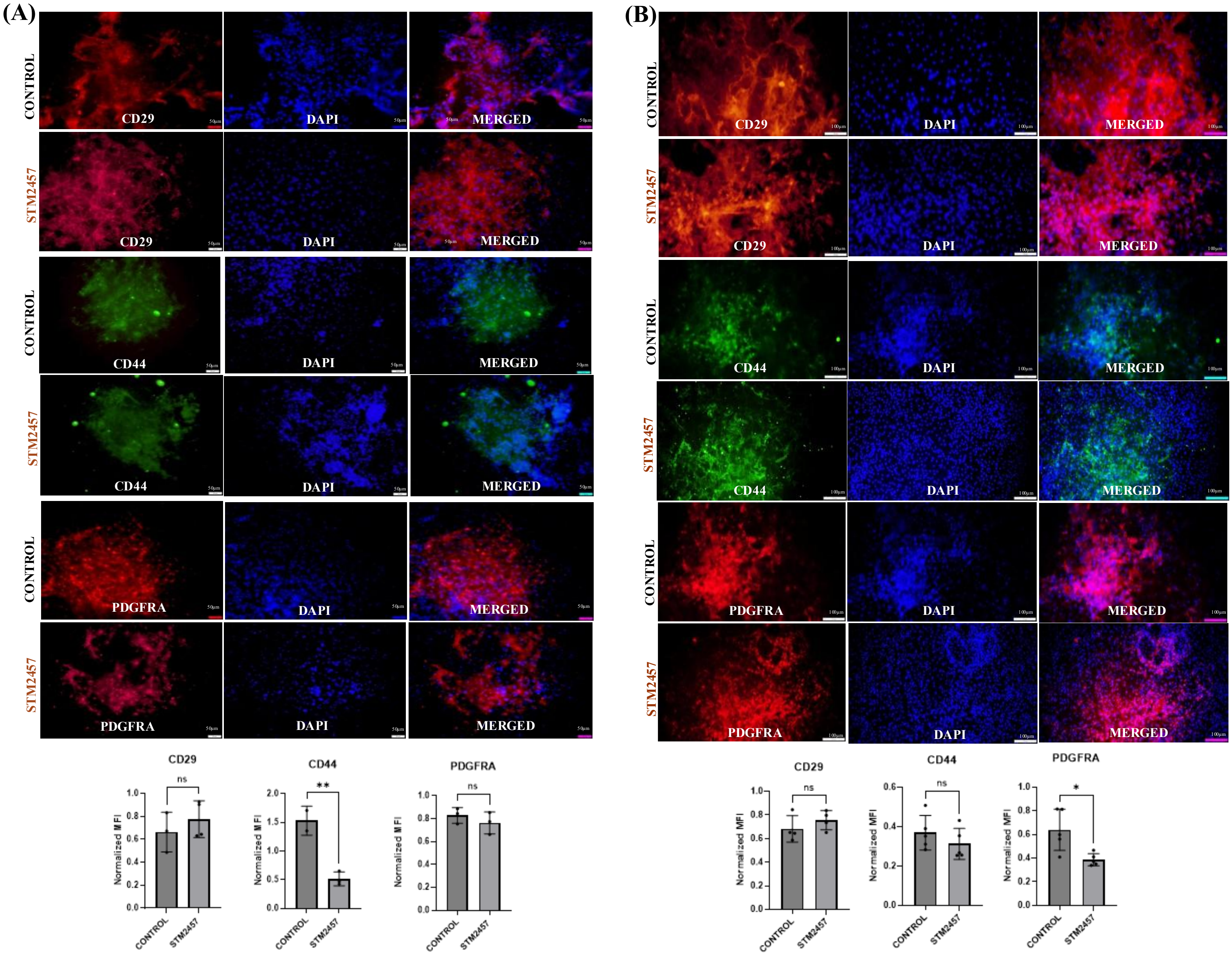

Supplementary Figure S3: Expression of adipose progenitor cell-surface markers on day 10 of beige adipogenesis of (A) HDFa-sourced iPSCs with STM2457 using IF; scale bar:50µm and (B) HDFn-sourced iPSCs with STM2457 using IF; scale bar:100µm. Expression analysis (mean fluorescence intensity) using ImageJ software are given below the images; Data represents mean ± SD across ≥ 4 fields of view from a single well per condition from a single iPSC line within one experiment. Individual data points represent fields of view; outliers removed. Statistical analysis was performed on averaged values per group using two-tailed unpaired t-test. Significance indicated as *p<0.05; ns, not significant. Representative images are shown for each well/group.

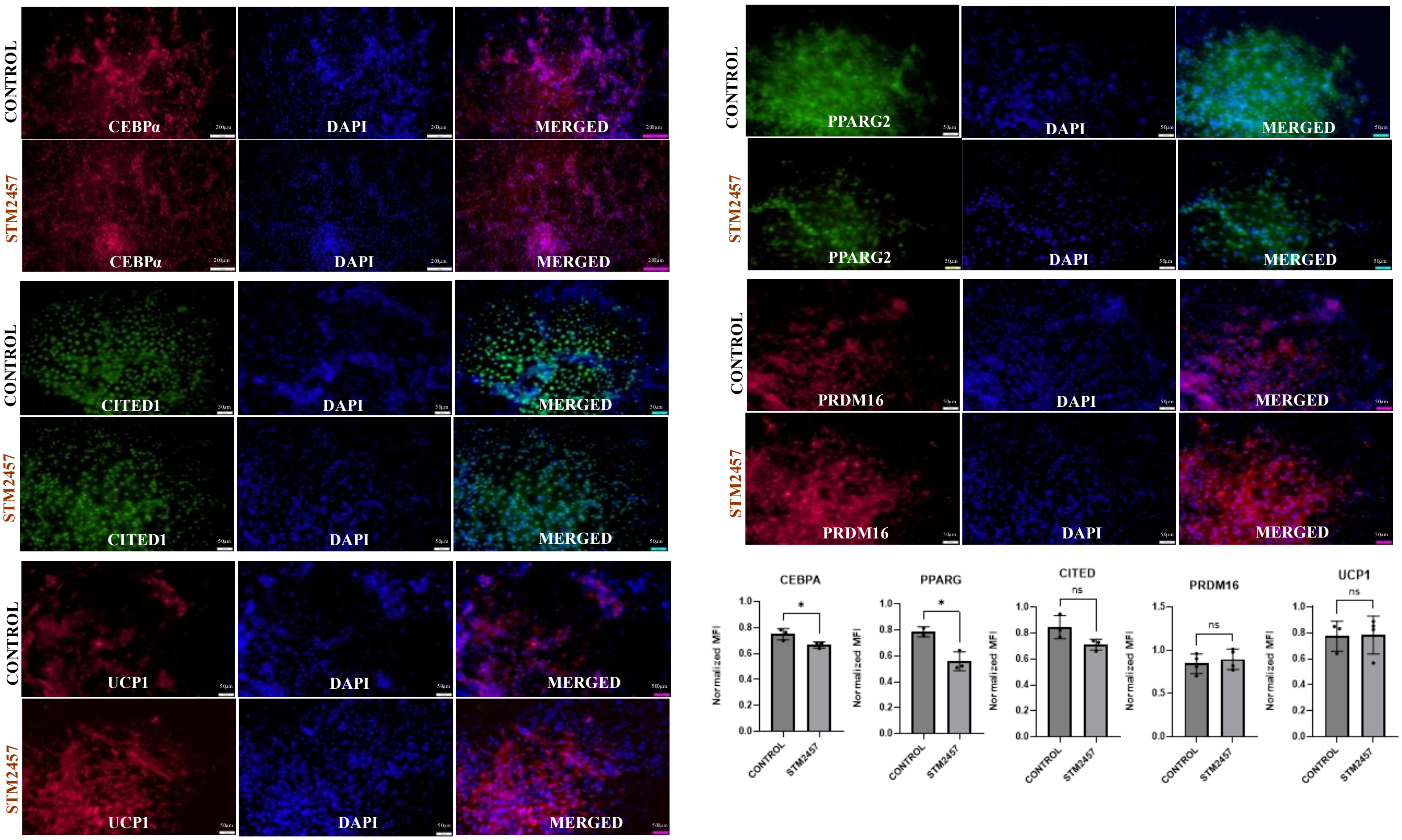

Supplementary Figure S4: Expression of the master adipogenic regulators and thermogenic adipocyte markers during beige adipogenesis of HDFa-sourced iPSCs with STM2457; scale bar: 50µm, (CEBPA 200 µm); Corresponding expression analysis (mean fluorescence intensity) using ImageJ software are given. Data represents mean ± SD across ≥ 4 fields of view from a single well per condition from a single iPSC line within one experiment. Individual data points represent fields of view; outliers removed. Statistical analysis was performed on averaged values per group using two-tailed unpaired t-test. Significance indicated as *$p<0.05$; ns, not significant. Representative images are shown for each well/group.

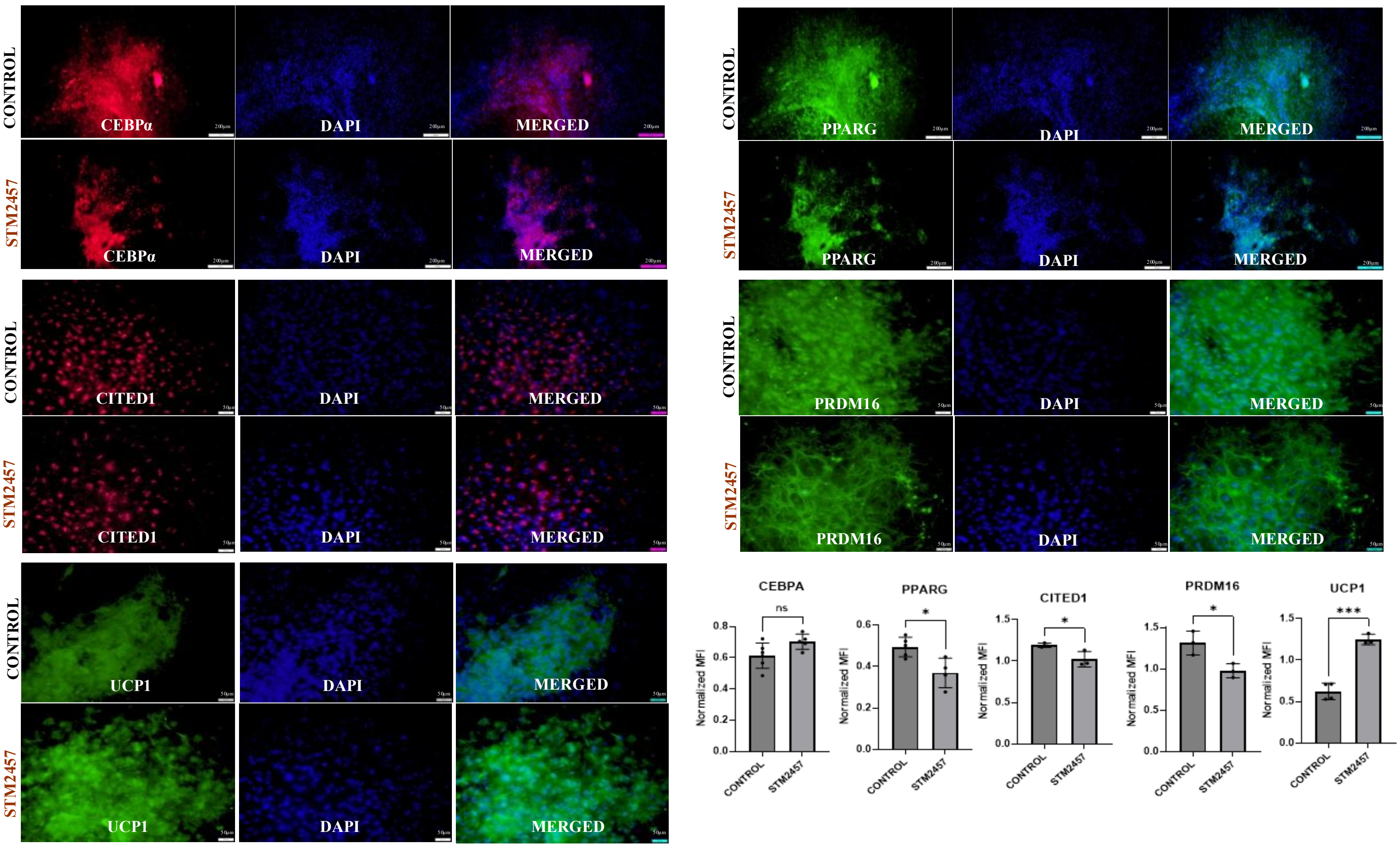

Supplementary Figure S5: Expression of the master adipogenic regulators and thermogenic adipocyte markers during beige adipogenesis of HDFn-sourced iPSCs with STM2457; scale bar: 50µm, (CEBPA and PPARG 200 µm); Corresponding expression analysis (mean fluorescence intensity) using ImageJ software are given. . Data represents mean ± SD across ≥ 4 fields of view from a single well per condition from a single iPSC line within one experiment. Individual data points represent fields of view; outliers removed. Statistical analysis was performed on averaged values per group using two-tailed unpaired t-test. Significance indicated as *p<0.05; ns, not significant. Representative images are shown for each well/group.

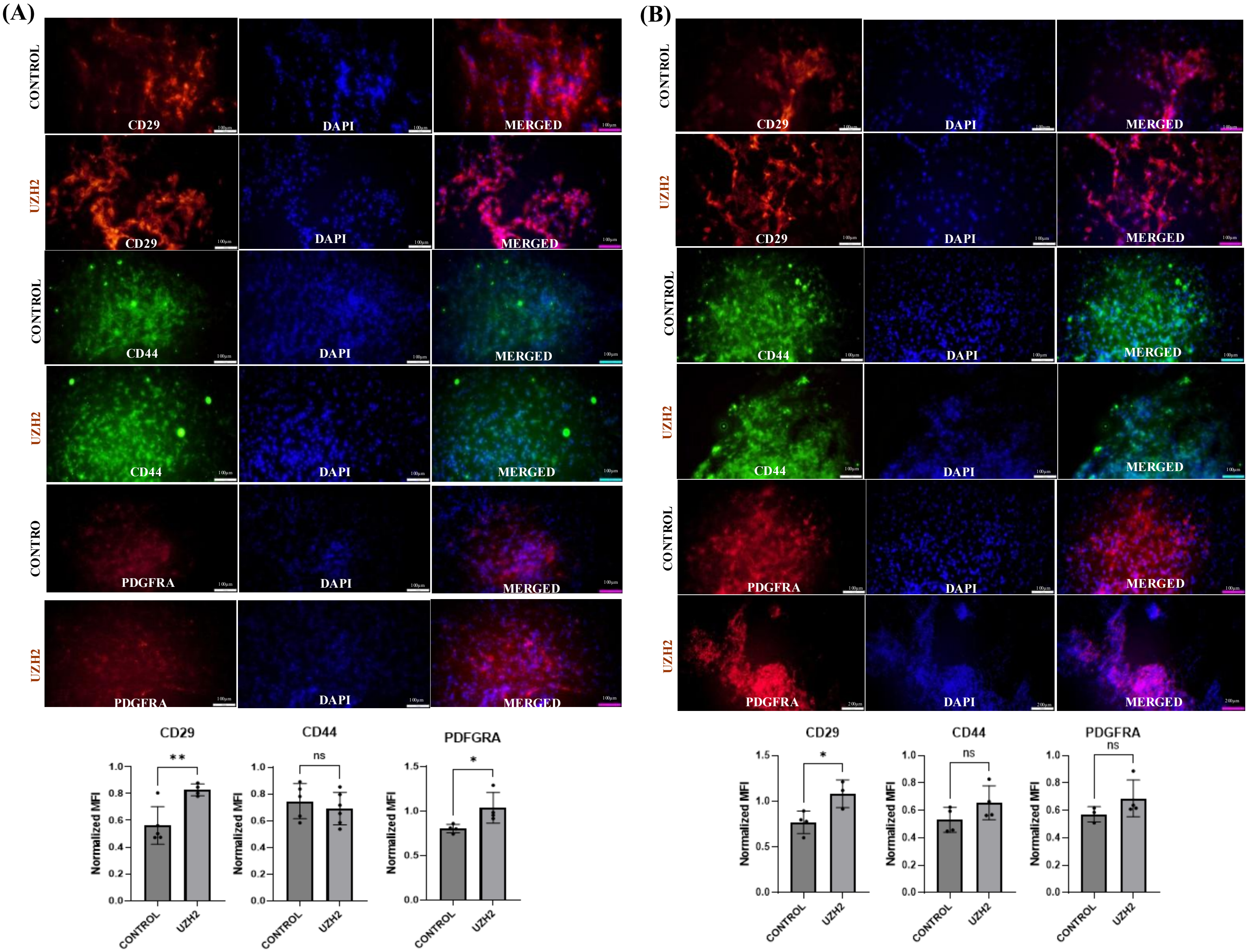

Supplementary Figure S6: Expression of adipose progenitor cell-surface markers on day 10 of beige adipogenesis of (A) HDFa-sourced iPSCs with UZH2 using IF; scale bar:100µm and (B) HDFn-sourced iPSCs with UZH2 using IF; scale bar:100µm (PDGFRA 200µm for UZH2). Expression analysis (mean fluorescence intensity) using ImageJ software are given below the images. Data represents mean ± SD across ≥ 4 fields of view from a single well per condition from a single iPSC line within one experiment. Individual data points represent fields of view; outliers removed. Statistical analysis was performed on averaged values per group using two-tailed unpaired t-test. Significance indicated as *p<0.05; ns, not significant. Representative images are shown for each well/group.

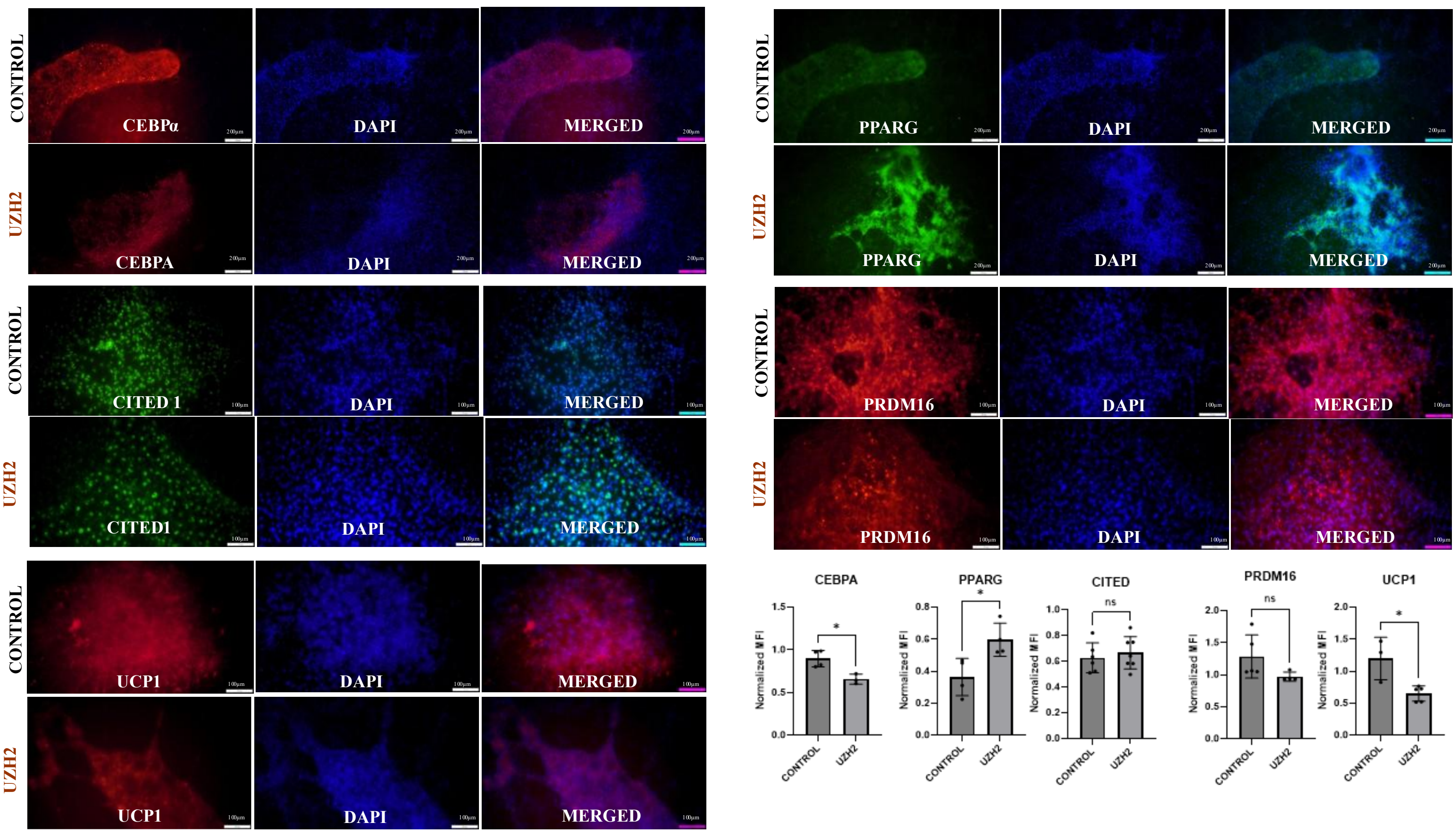

Supplementary Figure S7: Expression of the master adipogenic regulators and thermogenic adipocyte markers during beige adipogenesis of HDFa-sourced iPSCs with UZH2; scale bar: 100µm, (CEBPA and PPARG 200 µm); Corresponding expression analysis (mean fluorescence intensity) using ImageJ software are given. Data represents mean ± SD across ≥ 4 fields of view from a single well per condition from a single iPSC line within one experiment. Individual data points represent fields of view; outliers removed. Statistical analysis was performed on averaged values per group using two-tailed unpaired t-test. Significance indicated as *p<0.05; ns, not significant. Representative images are shown for each well/group.

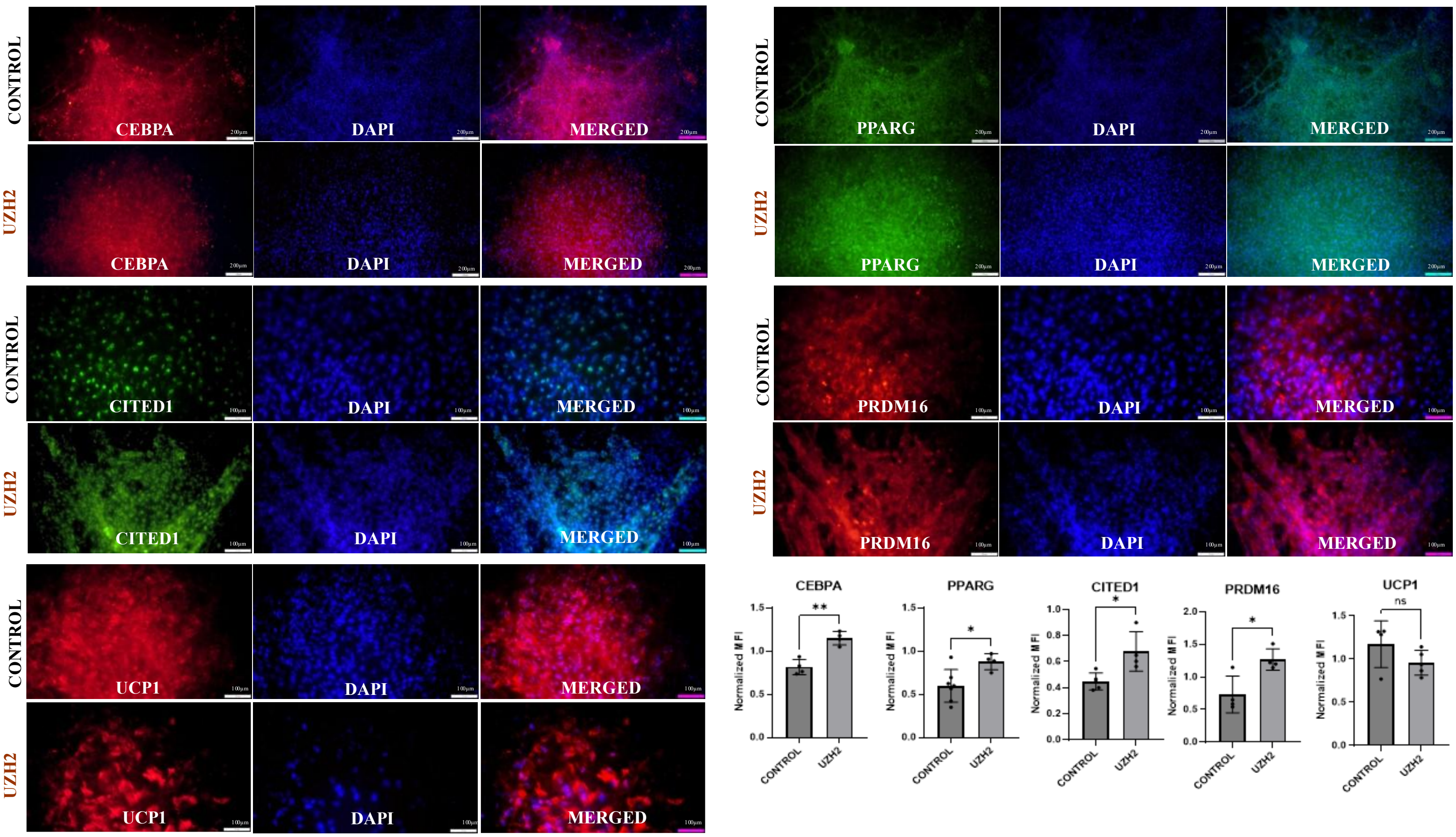

Supplementary Figure S8: Expression of the master adipogenic regulators and thermogenic adipocyte markers during beige adipogenesis of HDFn-sourced iPSCs with UZH2; scale bar: 100µm, (CEBPA and PPARG 200 µm); Corresponding expression analysis (mean fluorescence intensity) using ImageJ software are given. Data represents mean ± SD across ≥ 4 fields of view from a single well per condition from a single iPSC line within one experiment. Individual data points represent fields of view; outliers removed. Statistical analysis was performed on averaged values per group using two-tailed unpaired t-test. Significance indicated as *p<0.05; ns, not significant. Representative images are shown for each well/group.
